# Study protocol for fertility preservation discussions and decisions: A family-centered psychoeducational intervention for male adolescents and emerging adults newly diagnosed with cancer and their families

**DOI:** 10.1371/journal.pone.0263886

**Published:** 2022-02-16

**Authors:** Charis Stanek, Charleen I. Theroux, Anna L. Olsavsky, Kylie N. Hill, Joseph R. Rausch, Sarah H. O’Brien, Gwendolyn P. Quinn, Cynthia A. Gerhardt, Leena Nahata

**Affiliations:** 1 The Abigail Wexner Research Institute at Nationwide Children’s Hospital, Columbus, Ohio, United States of America; 2 The Ohio State University College of Medicine, Columbus, Ohio, United States of America; 3 Nationwide Children’s Hospital, Columbus, Ohio, United States of America; 4 New York University Grossman School of Medicine, New York, New York, United States of America; Public Library of Science, UNITED KINGDOM

## Abstract

Many childhood cancer survivors desire biological children but are at risk for infertility after treatment. One option for mitigating risk is the use of fertility preservation prior to gonadotoxic therapy. Adolescents and emerging adults may rely on their parents to help them decide whether to use fertility preservation. While this is often a collaborative process, it is currently unknown how parents can optimally support adolescents and emerging adults through this decision. To address this gap, we developed a family-centered, psychoeducational intervention to prompt adolescents and emerging adults to reflect on their future parenthood goals and attitudes towards fertility preservation, as well as to prompt their parents (or other caregivers) to reflect on their own and their child’s perspectives on the topic. In this randomized controlled trial, families will be randomized to either the standard of care control group (fertility consult) or the intervention group. After their fertility consult, adolescents and emerging adults and parents in the intervention group will complete a fertility preservation values clarification tool and then participate in a guided conversation about their responses and the fertility preservation decision. The primary expected outcome of this study is that participation in the intervention will increase the use of fertility preservation. The secondary expected outcome is an improvement in decision quality. Chi-square analyses and *t*-tests will evaluate primary and secondary outcomes. The goal of this intervention is to optimize family-centered fertility preservation decision-making in the context of a new cancer diagnosis to help male adolescents and emerging adults achieve their future parenthood goals.

## Introduction

Childhood cancer survival rates are on the rise, with 5-year survival rates now exceeding 80% [1,2]. As these rates increase, there is a growing number of children who will enter adulthood at-risk for a variety of late effects resulting from cancer treatment, including infertility [3]. Nearly 50% of male childhood cancer survivors experience fertility impairment as a result of adjuvant therapy [4–7], which can negatively impact quality of life and psychosocial functioning [8–13]. Many male adolescents and emerging adults (AEAs) with cancer desire biological parenthood; in a recent study, biological parenthood was viewed as a “top 3” life goal [14,15]. Sperm banking is a safe and effective pre-treatment fertility preservation (FP) method for AEA males to protect their ability to have a biological child in the future. However, only around 25% of pubertal males choose to bank sperm prior to treatment at many pediatric centers [11,16–19], which is concerning given that survivors often regret missed FP opportunities later in life [8–13].

The FP decision-making process can be challenging for AEAs and their families given the sensitivity of the topic, physical and psychological implications of a new cancer diagnosis, and the limited time to make FP decisions (sometimes as little as 12–24 hours) [17,20–23]. Parents are often unaware of their child’s future parenthood goals [24], withhold their own perspectives about FP [25], and frequently defer the FP decision to their son [26]. Given that AEAs are developmentally limited in their ability to engage in future-oriented thinking [27], research has shown that parents (especially fathers) play a key role in sperm banking decision-making [28–30]. Specifically, when parents encourage the use of FP, AEAs are more likely to attempt FP [29–31].

With the goal of facilitating FP decision-making during a new cancer diagnosis, our research team developed a novel family-centered FP values clarification tool (Family-centered Adolescent Sperm banking values clarification Tool, FAST) [32]. The tool was based on the Health Belief Model which illustrates how certain health beliefs and individual characteristics influence human behavior [33]. The Health Belief Model is incorporated into the intervention to assess key constructs, such as perceived risk for infertility, barriers to FP, and perceived benefits to FP. Research has shown that the Health Belief Model can be effective in predicting FP utilization [34,35], and sperm banking rates at our center doubled from 30% to 60% after implementation of this tool [32]. Further, when parents were concordant with their son’s fertility values and goals, AEAs were more likely to attempt FP [36]. One month after the FP decision, both FP attempters and non-attempters expressed short-term satisfaction with their decision, but FP non-attempters expressed the potential for future regret about the decision not to use FP [37].

These findings informed the development of our family-centered psychoeducational intervention, which involves offering a refined version of the FAST and a brief, facilitated family discussion based on their responses. The pilot randomized controlled trial (RCT) will evaluate feasibility, acceptability, and preliminary efficacy of this intervention, with the ultimate goal of optimizing FP utilization and improving decisional quality. The aim of this manuscript is to outline the study protocol for this pilot RCT.

## Methods

### Ethics

The Institutional Review Board (IRB) at a large pediatric academic medical center in the Midwest approved the study (#STUDY00000849) on 6/22/20. All parents and AEAs at least 18 years of age will provide written informed consent. AEAs under the age of the 18 will provide written assent. This trial is registered in ClinicalTrials.gov (Identifier: NCT04268004). Relevant data will be made available at the conclusion of this study and results from this trial will be submitted to ClinicalTrials.gov.

In addition to the NCH IRB, safety oversight for the study will be under the direction of a Data Safety Monitoring Board (DSMB). The DSMB will consist of two faculty members from NCH, who both share extensive experience in research with pediatric populations, as well as clinical trials. The DSMB will be responsible for safeguarding the interests of trial participants, assessing the safety and efficacy of the intervention(s) during the trial, and for monitoring the overall conduct of the clinical trial.

### Design

This is a single-site, randomized controlled trial (RCT) designed to test a FP decision-making intervention for AEA males newly diagnosed with cancer and their families. This study will take place at a large pediatric academic medical center in the Midwest. At this center, a fertility consult is automatically placed for all individuals newly diagnosed with cancer [38]. During the consult, the fertility specialist (advanced practice nurse or oncologist) provides information on infertility risk and reviews available FP options. Families in this study will be randomized (1:1) to either receive: 1) a standard of care fertility consult prior to cancer treatment, or 2) standard of care fertility consult prior to cancer treatment and the intervention. Families in both groups will be approached 1-month and 1-year after the initial visit to complete follow-up assessments. The objective of this RCT is to examine the feasibility of the intervention and preliminary efficacy of the intervention on FP attempt rates. The secondary objective of this RCT is to use a mixed methods approach to examine the effects of the intervention on FP decision quality.

We have two main hypotheses, which are:

1. Compared to the standard of care control group, male AEAs in the intervention group will have higher rates of FP utilization.
2. Families in the intervention group will report better quality family communication and higher decision quality compared to the control group.

### Sample size calculation

A sample size of *n* = 20 per group will provide 80% power to detect large effect sizes of *w* = .45 for the chi-square analysis and *d* = .91 for the independent samples *t*-test. Because this is a pilot study, the focus will be on generating preliminary effect size estimates for our primary outcome (FP utilization) comparing the intervention to the control group, rather than on statistical significance (e.g., using two-sided type I error rates of α < .05). The proposed sample size is sufficient for gleaning this information. The sample was determined based on the medium-large effects found in work by Klosky et al., which explored parent, provider, and AEA factors that influenced FP utilization [30].

### Setting and participants

Participants will include 40 families of 12- to 25-year-old males newly diagnosed with cancer at a large pediatric academic medical center in the Midwest. Parents and AEAs will be identified by the principal investigator, who is notified when a new fertility consult request is placed, or by the oncologists collaborating on the RCT. Eligible participants must be: (a) scheduled to receive chemotherapy and/or gonadal radiation, (b) pubertal (i.e., at least Tanner stage 2–3), and (c) proficient in English. Parents of eligible participants can include up to two parents or other primary parents (e.g., step-parent, grandparents) to be inclusive of different family structures and situations. If the AEA is less than 18 years of age, at least one parent or legal guardian must be present to provide consent for the AEA to participate.

### Recruitment and randomization

Following a fertility consult, research staff will recruit families in person (in either the inpatient or outpatient setting) or remotely (via phone). Research staff will explain the purpose of the study and ask the AEA and their parents (or other caregivers) if they are interested in participating. If they express interest, research staff will guide them through the informed consent process, provide them with a brief demographic survey, a follow-up form, and compensate each participating family member with a $5 meal card upon completion of measures. The research staff member will then exit the room, and the interventionist will enter to inform the family of their group assignment.

The interventionist will randomize each family through the data management software REDCap, which allows the randomization sequence generated by the statistician to be allocated via REDCap Randomization Module. In this module, the randomization sequence and group assignments are protected from the view of selected staff who use the REDCap program. Only the interventionist will have REDCap permissions to randomize and see the allocated group assignment when they log-on to REDCap. Study staff not involved in the intervention will be blinded to group assignment.

The REDCap program will have stratified randomization by age (<16 or ≥16) with blocks of 4 and 6 selected randomly (1:1) to either receive 1) standard of care fertility consult or 2) standard of care fertility consult and the intervention. The Center for Biobehavioral Health (CBH) Behavioral Trials Office (BTO) will maintain the randomization sequence. If the family is assigned to the control group, the first study visit is complete. If the family is assigned to the intervention group, they will complete the two-part intervention.

### Intervention

The family-centered intervention is based on the Health Belief Model and Family Systems Theory [39]. The Health Belief Model proposes that certain beliefs: perceived susceptibility, perceived severity, perceived benefits, perceived barriers, cues to action, and self-efficacy (Table 1) [40], play a key role in influencing health behavior change. The design of the FAST was informed by the Health Belief Model because the Health Belief Model has been shown to specifically predict FP [34,35]. This intervention expands on the FAST by incorporating guided conversation and parent perspectives, which can affect the FP decision-making process. Parent perspectives are especially important given that they play a key role in decision-making about FP [29–31,41,42]. The Family Systems Theory posits that individuals are connected to their family context, and each family member exerts an influence on each other’s behavior [43,44]. Thus, when AEAs make decisions about FP, the influence of the family must be considered in order to optimize decision-making [37,41,43,44]. The intervention addresses the importance of family-centered communication by prompting parents to reflect on their own FP knowledge and perspectives, as well as their son’s perspectives.

**Table 1:** Health Belief Model key concept definitions and applications.

Construct	Definition	Application
*Perceived Susceptibility*	A person’s belief in the chances of contracting an illness/experiencing a side effect	AEA’s belief in likelihood of experiencing infertility
*Perceived Severity*	A person’s belief about how intense the health problem will be	AEA’s belief in how severely infertility will affect their life
*Perceived Benefits*	A person’s belief about how a healthcare choice will diminish negative impact of an illness	AEA’s belief on how FP utilization can positively impact them
*Perceived Barriers*	A person’s belief about challenges associated with agreeing to a healthcare choice (cost, emotional impact, access, etc.)	AEA’s belief that certain barriers to FP utilization apply (cost, time, ability to produce a sample, etc.)
*Cues to Action*	A prompt to motivate a person into action	AEA being prompted to consider FP; being aware of FP as an option
*Self-Efficacy*	A person’s confidence in their capability of initiating the healthcare decision	AEA’s confidence in their capability of utilizing FP

In the first part of the intervention, the AEA and their parents complete the FAST. This survey takes approximately 5–10 minutes to complete and will be given electronically via REDCap. The AEA version will gather a self-report of thoughts and opinions about FP and future parenthood goals. The parent version will gather a self-report, as well as proxy report (i.e., parents will report on their perception of the AEA’s thoughts and perspectives). After the completion of this survey, the interventionist will immediately generate a report using a program that compares responses in REDCap. This report will highlight any similarities and differences in responses across each family member.

The intervention incorporates techniques from motivational interviewing, which has shown to be a useful tool in healthcare decision-making [37,45,46]. These techniques include rapport-building with the AEA and their parents, actively listening to their perspectives about FP, and outlining perceived benefits of FP to families. The interventionist will review the similarities and differences in perspectives between the parent(s) and the AEA as reported on the FAST, as well as areas of uncertainty or knowledge gaps. See Table 2 for an overview on the content addressed throughout the guided discussion. Pro-banking responses will be highlighted due to the time-sensitivity of the decision-making process and the documented experiences of regret from AEAs who decide not to bank [8–10,12]. Fathers’ perspectives will be highlighted before mothers’ because father input has been shown to exert more influence in the decision to pursue FP [29]. Since FP use has shown to be correlated with higher family-level concordance [36], this intervention integrates theory-driven decision-making tools that assist families in reaching concordance regarding FP knowledge and parenthood goals, in the hopes of increasing FP attempts. Afterwards, families will receive an email summarizing the content discussed throughout the guided discussion, as well as a copy of their FAST report generated from REDCap. The interventionist will send this email immediately following the discussion. The purposed of this email is to encourage families to continue the conversation about FP after the intervention has concluded, and to serve as a reference for them.

**Table 2:** Guided discussion protocol.

Steps	Content
1.	Highlight agreement or shared fertility-related values and goals in the family
2.	Highlight discrepancies between family members
3.	Highlight areas of uncertainty from parent about the AEA’s preferences
4.	Promote family communication and consensus building
5.	Reinforce known predictors of FP
6.	Address any knowledge gaps/misconceptions by referring families back to the fertility navigator for additional information

### Outcomes

To assess our variables of interest, we will collect data on demographic characteristics and outcome measures. Specifically, demographic characteristics will include age, race, ethnicity, religion, educational attainment, who they live with, relationship status, and income. Outcome measures include FP utilization and decision quality. Quantitative measures will be collected via paper or online REDCap surveys. Interviews will occur separately to allow each participant to speak freely. Interviews will be audio recorded, transcribed verbatim, and coded for thematic content by trained research staff.

**Fig 1:**
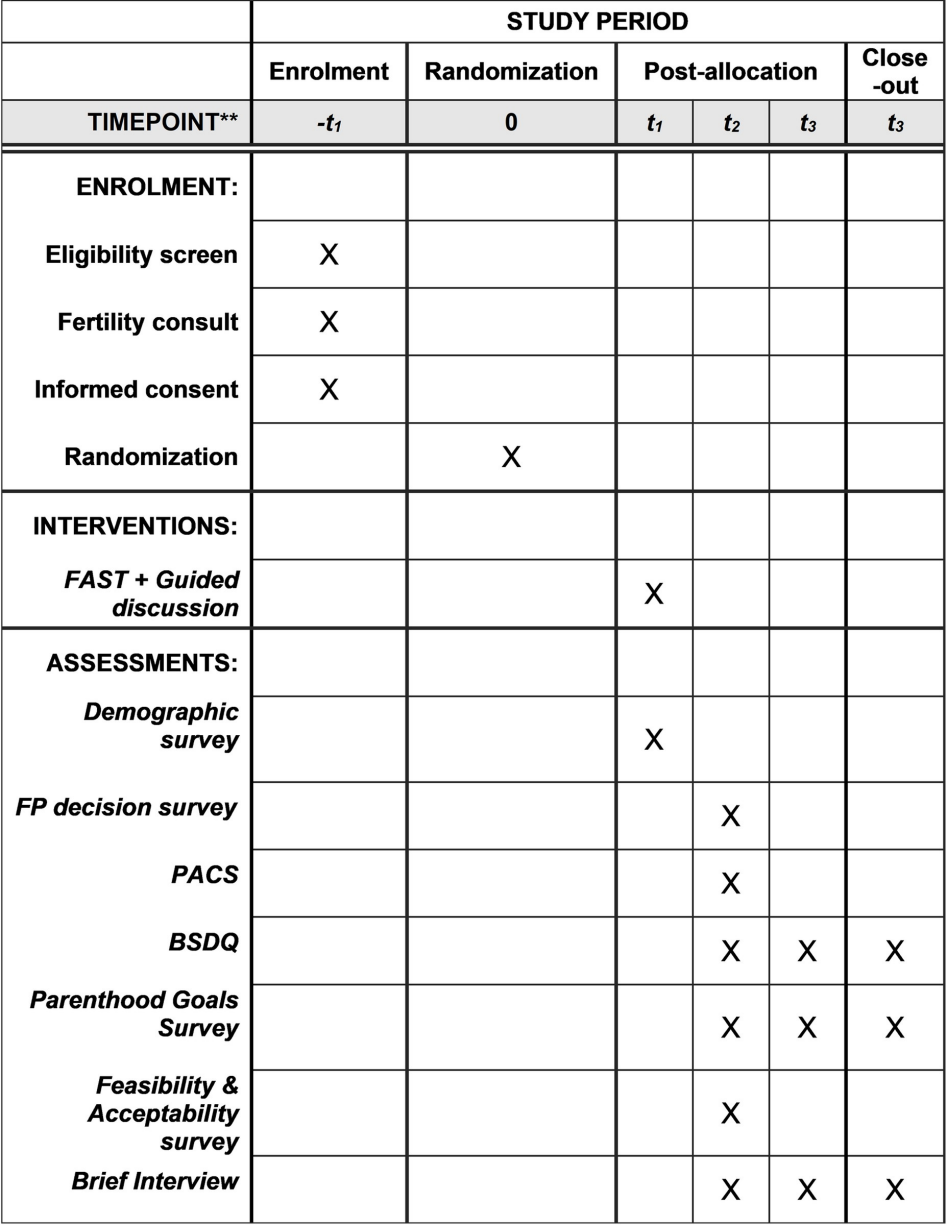
SPIRIT schedule describing study timeline.

After the first visit, families will be contacted either in person, by phone, or by email in one month and at one year for a follow-up assessment. In visit 2, families in the intervention group will complete the FP Decision Survey, the Parent-Adolescent Communication Scale (PACS), the Brief Subjective Decision Quality Measure (BSDQ), the Parenthood Goals Survey, the Feasibility and Acceptability Survey, and a brief interview. Families in the control group will complete only the FP Decision Survey, the PACS, the BSDQ, the Parenthood Goals Survey, and the brief interview. In visit 3, families in both the intervention and control group will complete the BSDQ, the Parenthood Goals Survey, and another brief interview. See Fig 1 for a timeline of the study. See Fig 1 for the study timeline. Measures are described in Table 3.

**Table 3:** Measure descriptions by visit.

Activity	Description
*Demographic Questionnaire*	Research staff ask participants to answer questions about age, race, ethnicity, religion, educational attainment, income (parents only), whom they live with, and their relationship to their child (parents only).
*Randomization*	Families will be randomized into one of two groups. If they are assigned to the control group, the study is complete. If they are assigned to the intervention group, they will participate in the intervention (step 3).
*Intervention (FAST + guided discussion)*	Participants will first complete the FAST and then participate in a guided conversation facilitated by the interventionist. Topics of the discussion include similarities and differences in perspectives between the AEA and their parents about FP, areas of uncertainty reported from parents about the AEA’s preferences, and both parent and AEA knowledge about the FP process.
*FP Decision Survey*	Participants will complete a brief survey that will prompt the participant to reflect on their FP decision. This survey will include questions such as who participants talked to about their FP decision and who made the final decision regarding FP.
*The Parent-Adolescent Communication Scale (PACS)*	A twenty-item scale used to measure perceived communication between AEAs and parents will be administered to both the AEA and their parents. AEAs will rate communication with both parents. Parents will only rate their communication with the AEA. Three scores are derived for openness, problems, and overall communication.
*Brief Subjective Decision Quality Measure (BSDQ)*	A six-item scale used to measure decision satisfaction administered to AEAs and parents. Items will be averaged into a composite decision satisfaction score (0–7).
*Parenthood Goals Survey*	A sixteen-item survey used to examine parental and AEA views on parenthood. This survey is a modified version of the FP Decision Tool. This survey will be given to families in both the control and intervention group.
*Feasibility and Acceptability Survey*	For the intervention group only, an eleven-item scale will be used to assess satisfaction with the intervention structure and content. The scale will include a final open-ended question asking for comments/suggestions about the intervention.
*Brief Interview*	The interview will focus on how participants made their FP decision, how they feel about the decision, and how the fertility counseling experience has impacted their family.

### Interventionist training and fidelity

The interventionist will have an advanced degree in a health-related field such as psychology, nursing, or public health. They will be trained in ENRICH/ECHO, which is an oncofertility communication training program [36] that consists of seven modules on the communication of reproductive health topics. The objective of the program is to help oncology health professionals effectively communicate key information regarding reproductive health to AEAs and their parents. The interventionist will also be trained on how to administer the FAST, create the REDCap report, and facilitate conversations between parents and AEAs. This training will involve a review of strategies for how to prioritize responses and how to engage in active listening.

**Fig 2:**
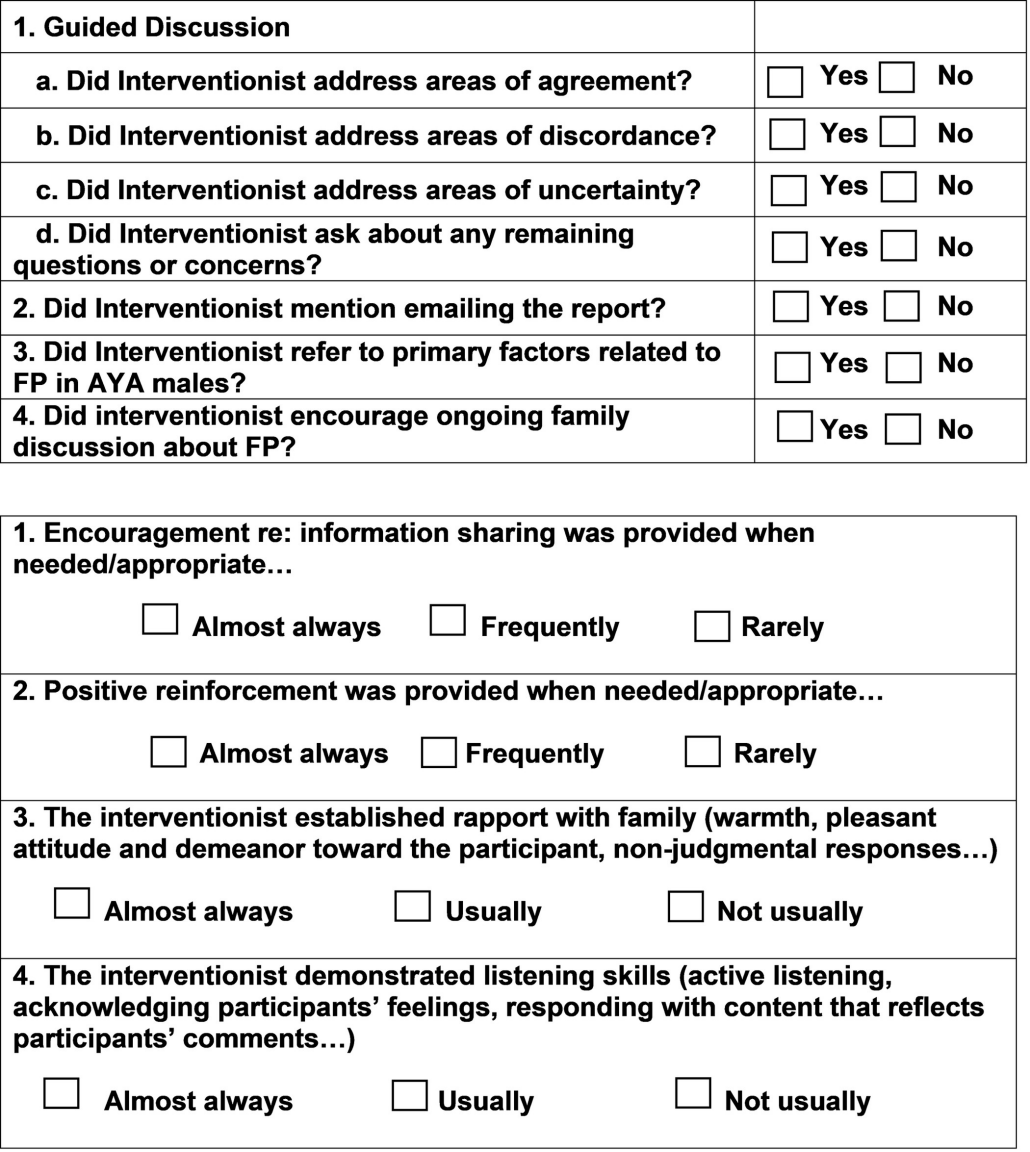
Fidelity checklist.

Every intervention session will be audio-recorded. Two principal investigators will independently complete a fidelity checklist (Fig 2) for each session to monitor consistency of intervention delivery and to ensure key points have been covered in the discussion.

## Statistical analyses

### Quantitative

Chi-square analyses and *t*-tests will be used to evaluate primary and secondary outcomes. A chi-square analysis will be used to examine the primary outcome of FP utilization (Y/N) as a function of being in the control or intervention group. An independent samples *t*-test will be used to examine whether the secondary outcomes of decision quality (numerical score on the Brief Subjective Decision Quality measure) are different between the groups at the 1-month and 1-year follow-ups.

The two groups (intervention and control) will be examined in an *exploratory* manner to assess potential differences in factors known to affect FP decisions. These factors include demographic characteristics (e.g., age), perceived time constraints, knowledge of FP, provider and parent recommendations, as well as novel factors, such as quality of parent-child communication. We will also examine these baseline factors for potential inclusion in an analysis of covariance to see if they might have a substantial impact on effect size estimates for the larger RCT.

### Qualitative

Recordings from the interview will be transcribed and imported into NVivo [47] (version 12) for analysis. Transcripts will be reviewed by a team of study staff. Thematic analysis will be used to identify common themes across interviews and to develop a category list. The technique employed will be the constant comparative analysis, in which interview content is compared across transcripts to reveal the most endorsed topics [48]. Multiple coders will be utilized to eliminate bias. The coding team will review transcripts in batches of 5–10 first for AEAs, until no new themes emerge. Once the coding team determines that no new themes are appearing, saturation will be reached. The coding team will repeat for interviews from mothers, and conclude with interviews from fathers. At this point, the coding team will review their tentative category lists and develop a finalized codebook, with definitions included. Next, study staff will use NVivo to code transcripts with the finalized themes and will identify frequency counts for each theme by participant. Kappa coefficients will be calculated using SPSS (version 26) to determine reliability amongst coders.

## Discussion

Previous research suggests that the implementation of a dedicated fertility program can improve FP utilization [49,50], yet families still face challenges in communicating and making decisions about FP. As a result, parents often rely on providers or external support to bridge these conversations and express communication barriers around FP [38]. AEAs with cancer face additional barriers regarding FP decision-making, including emotional exhaustion [51], feeling sick and disengaged in conversation, or feeling a sense of embarrassment around the topic of infertility [52]. Our previous research shows many families are open to communicating about FP decisions, but require guidance in facilitating these conversations [37]. Additionally, higher levels of family concordance on fertility perspectives is associated with increased FP utilization and improved decisional quality [36–38]. These findings and theoretical models, such as the Health Belief Model and Family Systems theory, have been instrumental in informing novel interventions to facilitate FP decision-making in pediatric oncology.

In this manuscript, we describe an RCT which will evaluate the feasibility, acceptability and preliminary efficacy of an intervention designed for male AEAs newly diagnosed with cancer, with the goal of optimizing FP utilization and decision quality. By using known factors in healthcare decision-making, in addition to newly identified factors of concordance and family communication, it is anticipated that this intervention will aid families in their decision-making process. At the completion of this study, we will be able to determine if the implementation of this intervention has an impact on FP utilization and decisional quality. To our knowledge, this is the first prospective, longitudinal study that tests the efficacy of an intervention guided by both the Health Belief Model and family systems theory on FP utilization. This body of work will address critical gaps in knowledge about how to optimize the FP decision-making process to help more adolescents and young adults successfully achieve their future parenthood goals.

## Supporting information

S1 File SPIRIT checklist. (PDF)

S2 File Study protocol. (DOCX)
