# Supplementary material for: Study protocol for fertility preservation discussions and decisions: A family-centered psychoeducational intervention for male adolescents and emerging adults newly diagnosed with cancer and their families
Source: PLoS One. 2022 Feb 16;17(2):e0263886. doi: 10.1371/journal.pone.0263886 (PMC8849538; doi:10.1371/journal.pone.0263886)
Supplement: S1 File — (PDF) [file pone.0263886.s001.pdf]

SPIRIT 2013 Checklist: Recommended items to address in a clinical trial protocol and related documents*

Section/item	Item No	Description
**Administrative information**		
Title	1	Study protocol for fertility preservation discussions and decisions: A family-centred psychoeducational intervention for male adolescents and emerging adults newly diagnosed with cancer and their families
Trial registration	2a	This trial is registered in ClincialTrials.gov (Identifier: NCT04268004)
Protocol version	3	V6, 23 October 2021

Original	
Revision #1	We updated information on the current status of our study, outline procedures for connecting a remote participant in, and updated the description of our surveys/methods to reflect changes made to those areas. We also changed the name of the study.
Revision #2	We updated information to include procedures for connecting an entire family in via Webex.
Revision #3	We included an additional questionnaire to give families during visit 2.
Revision #4	We updated our remote consent processes to include REDCap.
Revision #5	We included questionnaires to give to families during visit 3.

Funding	4	The Ohio State University Center for Clinical and Translational Science Voucher supported development of the web-based tool; The study is supported by The National Cancer Institute/Institutes of Health (K08CA237338)

Roles and responsibilities	5a	LN conceived of the study and CG, SB, GQ, and JR assisted with the study conceptualization. LN acquired funding for the project. LN, CG, SB, GQ, and JR contributed to the methodology. LN, AO, CT, KH, CS, and JR helped with project administration. LN, CG, SB, GQ, and JR supervised the project. LN, CS, AO, CT, and KH write the original draft, and CG, SB, GQ, and JR reviewed and edited the manuscript.
	5b	Dr. Nahata (Principal Investigator) is overseeing this trial at The Abigail Wexner Research Institute at Nationwide Children's Hospital. 700 Children's Dr. Columbus, OH 43205; 614-722-4502; leena.nahata@nationwidechildrens.org
	5c	The study sponsor and funders had no role in study design, collection, management, analysis, and interpretation of data; writing of the report; and the decision to submit the report for publication
	5d	**Principal Investigator**<br>Design of intervention<br>Preparation of protocols and revisions<br>Communication with Behavioral Trials Office<br>Publication of study reports<br>Oversight of data management team<br>Collaboration with clinical team<br><br>**Research Staff**<br>Data collection, entry, and management<br>Conducting intervention<br>Publication of study reports<br>Analysis of data

**Introduction**		
Background and rationale	6a	(See pages 3-4 in the manuscript.) Childhood cancer survival rates are on the rise, with 5-year survival rates now exceeding 80%.[1,2] As these rates increase, there is a growing number of children who will enter adulthood at-risk for a variety of late effects resulting from cancer treatment, including infertility.[3]<br><br>Existing Knowledge: Nearly 50% of male childhood cancer survivors experience fertility impairment as a result of adjuvant therapy,[4-7] which can negatively impact quality of life and psychosocial functioning.[8-13] Many male adolescents and emerging adults (AEAs) with cancer desire biological parenthood; in a recent study, biological parenthood was viewed as a "top 3" life goal.[14,15] Sperm banking is a safe and effective pre-treatment fertility preservation (FP) method for AEA males to protect their ability to have a biological child in the future. However, only around 25% of pubertal males choose to bank sperm prior to treatment at many pediatric centers,[11,16-19] which is concerning given that survivors often regret missed FP opportunities later in life.[8-13]<br><br>Need for a trial: The FP decision-making process can be challenging for adolescents and emerging adults (AEAs) and their families given the sensitivity of the topic, physical and psychological implications of a new cancer diagnosis, and the limited time to make FP decisions (sometimes as little as 12-24 hours).[17,20-23] Parents are often unaware of their child's future parenthood goals[24], withhold their own perspectives about FP[25], and frequently defer the FP decision to their son.[26] Given that AEAs are developmentally limited in their ability to engage in future-oriented thinking[27], research has shown that parents (especially fathers) play a key role in sperm banking decision-making.[28-30] Our previous studies showed that when parents were concordant with their son's fertility values and goals, AEAs were more likely to attempt FP[31] and that FP non-attempters expressed the potential for future regret about the decision not to use FP.[32] These findings informed the development of our family-centered psychoeducational intervention, which involves offering a refined version of the FAST and a brief, facilitated family discussion based on their responses. The pilot randomized controlled trial (RCT) will evaluate feasibility, acceptability, and preliminary efficacy of this intervention, with the ultimate goal of optimizing FP utilization and improving decisional quality.
	6b	Participants in the control group will receive a standard of care fertility consult in order to test the efficacy of the intervention compared to standard care.
Objectives	7	The study aims are to assess feasibility, acceptability and efficacy of a novel family-centered psychoeducational FP intervention in male AEAs newly diagnosed with cancer. The two main hypotheses are:<br>1) Compared to the standard of care control group, male AEAs in the intervention group will have higher rates of FP utilization.<br>2) Families in the intervention group will report better quality family communication and higher decision quality compared to the control group.

Trial design	8	The family-centered psychoeducational intervention is designed as a randomized and controlled trail. Participants will be randomized by the data management software REDCap. The REDCap program will have stratified randomization by age (<16 or ≥16) with blocks of 4 and 6 selected randomly (1:1) to either receive 1) standard of care fertility consult or 2) standard of care fertility consult and the intervention. The Center for Biobehavioral Health (CBH) Behavioral Trials Office (BTO) will maintain the randomization sequence.

Methods: Participants, interventions, and outcomes

Study setting	9	(See page 4 in the manuscript.) Participants will include 40 families of 12- to 25-year-old males newly diagnosed with cancer at a large pediatric academic medical center in the Midwest (Nationwide Children's Hospital).
Eligibility criteria	10	Eligible participants must be: (a) scheduled to receive chemotherapy and/or gonadal radiation, (b) pubertal (i.e., at least Tanner stage 2-3), and (c) proficient in English. Parents of eligible participants can include up to two parents or other primary parents (e.g., step-parent, grandparents) to be inclusive of different family structures and situations. If the AEA is less than 18 years of age, at least one parent or legal guardian must be present to provide consent for the AEA to participate. Participants are not eligible if they have previously received chemotherapy, have not reached Tanner stage 2-3, are not fluent in English, or have a cognitive impairment or developmental disability that would impact their ability to participate in a guided conversation facilitated by the interventionist (a trained research assistant or post-doc).
Interventions	11a	(See pages 8-10 in the manuscript.) The interventionist will randomize each family through the data management software REDCap, which allows the randomization sequence generated by the statistician to be allocated via REDCap Randomization Module. If the family is assigned to the control group, the first study visit is complete. If the family is assigned to the intervention group, they will complete the two-part intervention. In the first part of the intervention, the AEA and their parents complete the FAST. This survey takes approximately 5-10 minutes to complete and will be given electronically via REDCap. The AEA version will gather a self-report of thoughts and opinions about FP and future parenthood goals. The parent version will gather a self-report, as well as proxy report (i.e., parents will report on their perception of the AEA's thoughts and perspectives). After the completion of this survey, the interventionist will immediately generate a report using a program that compares responses in REDCap. This report will highlight any similarities and differences in responses across each family member.<br><br>The intervention incorporates techniques from motivational interviewing, which has shown to be a useful tool in healthcare decision-making.[32-34] These techniques include rapport-building with the AEA and their parents, actively listening to their perspectives about FP, and outlining perceived benefits of FP to families. The interventionist will review the similarities and differences in perspectives between the parent(s) and the AEA as reported on the FAST, as well as areas of uncertainty or knowledge gaps.
	11b	NA
	11c	NA

11d Standard of care fertility consults will be provided to participants in both the control and intervention arm. No interventions are prohibited.

Outcomes	12	(See pages 10-12 in the manuscript.) Primary outcome measures:<br>• Fertility Preservation (FP) Uptake [Time Frame: Baseline to before AEA begins treatment (generally within one to two weeks from baseline)]<br><br>Secondary outcome measures:<br>1. Brief Subjective Decision Quality (BSDQ) Questionnaire - AEA [Time Frame: 1-month post randomization + 1-year post randomization]<br><br>AEAs will complete the six-item Brief Subjective Decision Quality (BSDQ) Questionnaire with total scores ranging from 1-7; higher scores indicate greater satisfaction with their decision.<br><br>2. Brief Subjective Decision Quality (BSDQ) Questionnaire - Caregiver [Time Frame: 1-month post randomization + 1-year post randomization]<br><br>Caregivers will complete the six-item Brief Subjective Decision Quality (BSDQ) Questionnaire with total scores ranging from 1-7; higher scores indicate greater satisfaction with their decision.<br><br>Other outcomes:<br><br>3. FP Decision Survey – AEA [Time Frame: 1-month post randomization + 1-year post randomization]<br><br>AEAs will complete a brief survey that will prompt them to reflect on their FP decision. This survey will include questions such as who they talked to about their FP decision and who made the final decision regarding FP.<br><br>4. FP Decision Survey – AEA [Time Frame: 1-month post randomization + 1-year post randomization]<br><br>Caregivers will complete a brief survey that will prompt them to reflect on their FP decision. This survey will include questions such as who they talked to about their FP decision and who made the final decision regarding FP.<br><br>5. Parenthood Goals Survey - AEA [Time Frame: 1-month post randomization + 1-year post randomization]<br><br>AEAs will complete a sixteen-item survey used to examine parental and AEA views on parenthood. This survey is a modified version of the FP Decision Tool. This survey will be given to AEAs in both the control and intervention group.<br><br>6. Parenthood Goals Survey - Caregiver [Time Frame: 1-month post randomization + 1-year post randomization]<br><br>Caregivers will complete a sixteen-item survey used to examine parental and AEA views on parenthood. This survey is a modified version of the FP Decision Tool. This survey will be given to caregivers in both the control and intervention group.<br><br>7. Parent Adolescent Communication Scale (PACS) - AEA [Time Frame: 1-month post-randomization]<br><br>AEAs will complete the twenty-item Parent Adolescent Communication Scale (PACS), with total scores ranging from 20-100 and subscales (openness and problems) ranging from 10-50. Higher total and openness scores indicate better communication; higher problems scores indicate worse communication.

8. Parent Adolescent Communication Scale (PACS) - Caregiver
[Time Frame: 1-month post-randomization]

Caregivers will complete the twenty-item Parent Adolescent Communication Scale (PACS), with total scores ranging from 20-100 and subscales (openness and problems) ranging from 10-50. Higher total and openness scores indicate better communication; higher problems scores indicate worse communication.

9. Feasibility and Acceptability Survey – AEA [Time Frame: 1-month post-randomization]

For the intervention group only, AEAs will complete an eleven-item scale will be used to assess satisfaction with the intervention structure and content. The scale will include a final open-ended question asking for comments/suggestions about the intervention.

10. Feasibility and Acceptability Survey – Caregiver [Time Frame: 1-month post-randomization]

For the intervention group only, Caregivers will complete an eleven-item scale will be used to assess satisfaction with the intervention structure and content. The scale will include a final open-ended question asking for comments/suggestions about the intervention.

Participant timeline | 13 | (See page 11 in the manuscript.) Figure 1. Schedule of enrollement, interventions, and assessments.

	STUDY PERIOD					
	**Enrolment**	**Randomization**	**Post-allocation**			**Close -out**
**TIMEPOINT****	**-t_1**	**0**	**t_1**	**t_2**	**t_3**	**t_3**
**ENROLMENT:**						
**Eligibility screen**	X					
**Fertility consult**	X					
**Informed consent**	X					
**Randomization**		X				
**INTERVENTIONS:**						
FAST + Guided discussion			X			
**ASSESSMENTS:**						
Demographic survey			X			
FP decision survey				X		
PACS				X		
BSDQ				X	X	X
Parenthood Goals Survey				X	X	X
Feasibility & Acceptability survey				X		
Brief Interview				X	X	X

Sample size	14	(See pages 7-8 in the manuscript.) A sample size of *n*=20 per group will provide 80% power to detect large effect sizes of *w*=.45 for the chi-square analysis and *d*=.91 for the independent samples *t*-test. Because this is a pilot study, the focus will be on generating preliminary effect size estimates for our primary outcome (FP utilization) comparing the intervention to the control group, rather than on statistical significance (e.g., using two-sided type I error rates of α < .05). The proposed sample size is sufficient for gleaning this information. The sample was determined based on the medium-large effects found in work by Klosky et al., which explored parent, provider, and AEA factors that influenced FP utilization.[30]
Recruitment	15	Research staff will screen potentially eligible participants using the hospital's electronic medical record. The principal investigator is in direct contact with the oncofertility team and will receive notification when new fertility consults have been placed for this population. Screening will continue until the target population is achieved (40 families). The enrollment period will extend over two years, or until our desired sample size is enrolled. Each subject will receive a $5 meal card for their participation in the intervention.

Methods: Assignment of interventions (for controlled trials)

Allocation:

Sequence generation	16a	(See pages 5-6 in the manuscript.) Participants will be randomly assigned to either control or experimental group with a 1:1 allocation by computer generated randomization stratified by age (<16 or ≥16) with blocks of 4 and 6 selected randomly (1:1) to either receive 1) standard of care fertility consult or 2) standard of care fertility consult and the intervention.
Allocation concealment mechanism	16b	Participants will be randomized using REDCap, which is a data management software program in which randomization sequences can be added to survey design. Allocation will be concealed until the participant is recruited into the trial and has completed the demographic questionnaire.
Implementation	16c	All patients who complete the consent process for the study will be randomized. Randomization will be completed by a trained interventionist on the study. Once the demographic questionnaire is completed, the interventionist will randomize the participant and proceed from there based on group assignment. Staff responsible for recruitment will not be informed about the group allocation. Participants will be assigned to groups based on a sequence generated by REDCap, stratified by age with blocks of 4 and 6 selected randomly to be assigned to the control or intervention group. The Behavioral Trials Office in the Center for Biobehavioral Health will maintain the randomization sequence. Principal investigators and recruitment staff will have no influence on the randomization.

Blinding (masking)	17a	The randomization sequence and group assignments are protected from the view of selected staff who use the REDCap program. Only the interventionist will have REDCap permissions to randomize and see the allocated group assignment when they log-on to REDCap. Study staff not involved in the intervention will be blinded to group assignment. Due to the design of the intervention, participants cannot be blinded to their group assignment. The interventionist will make sure not to disclose the group assignments to other research staff by spending 15 minutes in the hospital after allocation when they are in the control group in order not to reveal the group allocation by returning early. Other research staff will not have access to any documentation with participant names or other identifying information that is paired with group assignments.
	17b	There is no circumstance in which knowledge of group assignment is essential for research staff other than the interventionist. Due to the nature of the intervention, there would be no reason for a participant to need to be assigned to a new group. If a participant wished to be taken out of the intervention group, they could withdraw from the study, but would not be assigned to the control group as a result.

Methods: Data collection, management, and analysis

Data collection methods	18a	(See pages 10-12 in the manuscript.) In the first part of the intervention, the AEA and their parents complete the FAST. The AEA version will gather a self-report of thoughts and opinions about FP and future parenthood goals. The parent version will gather a self-report, as well as proxy report (i.e., parents will report on their perception of the AEA's thoughts and perspectives). After the completion of this survey, the interventionist will immediately generate a report using a program that compares responses in REDCap. This report will highlight any similarities and differences in responses across each family member. The interventionist will review the similarities and differences in perspectives between the parent(s) and the AEA as reported on the FAST, as well as areas of uncertainty or knowledge gaps. To assess our variables of interest, we will collect data on demographic characteristics and outcome measures. Specifically, demographic characteristics will include age, race, ethnicity, religion, educational attainment, who they live with, relationship status, and income. Outcome measures include FP utilization and decision quality. Measures will be collected via paper or online REDCap surveys.<br><br>Participants will complete the Parent-Adolescent Communication Scale (PACS) at visit 2. A twenty-item scale used to measure perceived communication between AEAs and parents will be administered to both the AEA and their parents. AEAs will rate communication with both parents. Parents will only rate their communication with the AEA. Three scores are derived for openness, problems, and overall communication. The Cronbach's alpha is 0.87 for openness and 0.78 for the problems subscale. In visits 2 and 3, participants will complete the Brief Subjective Decision Quality measure. This is a six-item scale used to measure decision satisfaction administered to AEAs and parents. Items will be averaged into a composite decision satisfaction score (0-7). The Cronbach's alpha for this scale was 0.78 overall. In visit 2 and 3, participants will also complete the Parenthood Goals Survey. This is a sixteen-item survey used to examine parental and AEA views on parenthood. This survey is a modified version of the FP Decision Tool. This survey will be given to families in both the control and intervention group. In visit 3 only participants will complete the Feasibility and Acceptability survey, designed specifically for this study. This survey is completed by participants in the intervention group only, and is an eleven-item scale will be used to assess satisfaction with the intervention structure and content. The scale will include a final open-ended question asking for comments/suggestions about the intervention. Participants will also be briefly interviewed in visits 2 and 3, which will be audio recorded and transcribed for analysis.

	18b	Once a family enrolls in the study, study staff will make every effort to follow up with the family for the entire study period to maintain enrollment. Study staff will follow-up with participants in clinic at the 1-month and 1-year follow-up dates. If study staff approach at a time that is not convenient for the participant, they will coordinate a better time with the family to return. If the family is not expected to be in-clinic around the 1-month or 1-year follow-up, families will be contacted via phone. Study staff will attempt to approach families in follow-up visits for the duration of the study or until a family decides to withdraw from the study. A participant will be considered lost to follow-up if they fail to return for scheduled visits and study staff are unable to contact the participant after at least 3 attempts via phone or email. Participants can withdraw from the study at any point in time. Study staff reach out to participants with holiday cards each year as an effort to maximize retention. Study staff also review responses from the *Feasibility and Acceptability Survey* to understand how to improve our intervention and enhance participants' overall experience in the study.
Data management	19	(See page 7in the manuscript.) Data will be collected by trained research staff using an online survey tool (e.g., REDCap) or paper and pen questionnaires. Some data may be obtained from the participant's electronic medical record. Data collected at Visit 1 will be collected before the intervention begins, and data collected at Visits 2 and 3 will be collected after the intervention ends. Online questionnaire data will be housed behind a secure firewall on the NCH research internet server. Electronic recordings will be stored on NCH computers. Data collected on paper will be entered into electronic study databases and original files will be kept in a locked cabinet within a locked office at the hospital. Only direct study personnel will have access to this information. Study documents will be retained for a minimum of 2 years after the formal discontinuation of clinical development of the study intervention. These documents will be retained for a longer period, if required by local regulations.

Statistical methods	20a	(See pages 13 in the manuscript.) Chi-square analyses and *t*-tests will be used to evaluate primary and secondary outcomes. A chi-square analysis will be used to examine the primary outcome of FP utilization (Y/N) as a function of being in the control or intervention group. An independent samples *t*-test will be used to examine whether the secondary outcomes of decision quality (numerical score on the Brief Subjective Decision Quality measure) are different between the groups at the 1-month and 1-year follow-ups.<br><br>The two groups (intervention and control) will be examined in an *exploratory* manner to assess potential differences in factors known to affect FP decisions. These factors include demographic characteristics (e.g., age), perceived time constraints, knowledge of FP, provider and parent recommendations, as well as novel factors, such as quality of parent-child communication. We will also examine these baseline factors for potential inclusion in an analysis of covariance to see if they might have a substantial impact on effect size estimates for the larger RCT.<br><br>Recordings from the interview will be transcribed and imported into NVivo[35] (version 12) for analysis. Transcripts will be reviewed by a team of study staff. Thematic analysis will be used to identify common themes across interviews and to develop a category list. The technique employed will be the constant comparative analysis, in which interview content is compared across transcripts to reveal the most endorsed topics.[36] Multiple coders will be utilized to eliminate bias. The coding team will review transcripts in batches of 5-10 first for AEAs, until no new themes emerge. Once the coding team determines that no new themes are appearing, saturation will be reached. The coding team will repeat for interviews from mothers, and conclude with interviews from fathers. At this point, the coding team will review their tentative category lists and develop a finalized codebook, with definitions included. Next, study staff will use NVivo to code transcripts with the finalized themes and will identify frequency counts for each theme by participant. Kappa coefficients will be calculated using SPSS (version 26) to determine reliability amongst coders.
	20b	NA
	20c	Participants may be withdrawn if the intervention causes distress and it is determined by the interventionist and/or medical team that is not in the participant’s best interest to continue. If the participant withdrawals from the intervention, study staff will encourage completion of study data collection, per intent-to-treat methodology. Should participants withdraw after randomization, sensitivity analyses will be completed to assess potential differences in focal analyses by whether or not participants withdrew during the study. Missing data will be assessed and handled on a case by case basis using modern missing data analytic techniques (e.g., prorated scoring, multiple imputation, full information maximum likelihood, etc.).

Methods: Monitoring

Data monitoring	21a	(See pages 4-5 in the manuscript.) Safety oversight for the study will be under the direction of the NCH IRB, as well as a Data Safety Monitoring Board (DSMB). The DSMB will consist of two faculty members from NCH. The first is a Professor of Pediatrics and Psychology at The Ohio State University and Director of the Biobehavioral Outcomes Core at NCH. She is a leading researcher in psychosocial outcomes among children and adolescents with significant health challenges, who has experience developing and executing intervention research with pediatric populations, as well as conducting multi-site studies involving complex multi-method longitudinal assessments. The second is an Associate Professor at The Ohio State University, an attending physician in Hematology/Oncology at NCH, director of the AYA Oncology Program, and a member of the Fertility and Reproductive Health team at NCH. He has been heavily involved in clinical research through the Children's Oncology Group, specifically in the enrollment and management of patients on clinical trials.<br><br>The DSMB will be responsible for safeguarding the interests of trial participants, assessing the safety and efficacy of the intervention(s) during the trial, and for monitoring the overall conduct of the clinical trial. The DSMB will periodically review study results, evaluate the study interventions and procedures for adverse effects, and judge whether the overall integrity and conduct of the trial remain acceptable. Moreover, the DSMB may formulate and put forth recommendations to the study team, relating to the selection/recruitment/retention of participants and their management, and compliance to protocol specified regimens and the procedures for data management and quality control.
	21b	Summary reports regarding number of enrolled participants, and safety and risk (including any adverse events and unanticipated problems), will be generated at six-month intervals for the DSMB. Adverse events will be reported to the DSMB and IRB when they occur to determine if new procedures need to be implemented.<br><br>Safety information will be collected during routine interactions (phone calls, in-person visits, data collection timepoints) with participants.<br><br>Once the study begins, the study team will meet weekly to review the progress of the study (including information such as recruitment rate, reasons for declining participation, reasons for attrition), review data entry, assure data accuracy, detect potential errors at an early stage, and monitor staff compliance with training, regulatory, and confidentiality procedures. The research will be suspended if the DSMB determines that is the best course of action, based on the data provided for review.

Harms 22 This protocol defines an adverse event (AE) as any negative physical or emotional condition that was not present at baseline. *This protocol defines a serious adverse event (SAE) as any AE that results in hospitalization, permanent disability, or death.*

Severity: All AEs and SAEs are assessed by the principal investigator, and if necessary, another professional with clinical experience in the study population to determine their severity.

For AEs and SAEs, the following guidelines are used to describe severity:

- Mild: Events require minimal or no treatment and do not interfere with the participant's daily activities.
- Moderate: Events result in a low level of inconvenience or concern with the therapeutic measures. Moderate events may cause some interference with functioning.
- Severe: Events interrupt a participant's usual daily activity and may require systemic drug therapy or other treatment. Severe events are life-threatening or incapacitating.

Relationship to the Intervention: All AEs and SAEs are assessed by the principal investigator, and if necessary, another professional with clinical experience in the study population to determine their relationship to study intervention. The evaluation of relatedness considers etiologies such as natural history of the underlying disease, concurrent illness, concomitant therapy, study-related procedures, accidents, and other external factors.

The degree of certainty about causality is graded using the following categories:

- Related: The event is known to occur with the study procedures, there is a reasonable possibility that the study procedures caused the event, or there is a temporal relationship between the study procedures and the event. Reasonable possibility means that there is evidence to suggest a causal relationship between the study procedures and the event.
- Not Related: There is not a reasonable possibility that the study procedures caused the event, there is no temporal relationship between the study procedures and event onset, or an alternate etiology has been established.

AEs and SAEs are captured on the appropriate case report form (CRF). Information collected includes event description; time of onset; clinician assessment of severity, relationship to study procedures (assessed only by those with the training and authority to make a diagnosis), expectedness; and time of resolution/stabilization of the event. All AEs and SAEs occurring during the above-mentioned timeframe are documented appropriately regardless of relationship to the intervention. Events are followed for outcome information until resolution or stabilization.

Auditing	23	The study team will review protocol deviations on an ongoing basis and will implement corrective actions when the quantity or nature of deviations are deemed to be at a level of concern. This protocol defines a protocol deviation as any noncompliance with the clinical trial protocol, International Council on Harmonization Good Clinical Practice (ICH GCP), or Manual of Procedures (MOP) requirements. The noncompliance may be either on the part of the participant, the investigator, or the study site staff. As a result of deviations, corrective actions will be developed by the site and implemented promptly, if needed.<br><br>It is the responsibility of the PI to use continuous vigilance to identify and report deviations as required by the IRB and the funder, as well as oversee quality control of data. The site investigator is responsible for knowing and adhering to the reviewing IRB requirements. Should independent monitoring become necessary, the PI will provide direct access to all trial related sites, source data/documents, and reports for the purpose of monitoring and auditing by the funding agency, and inspection by local and regulatory authorities.

Ethics and dissemination

Research ethics approval	24	The protocol, informed consent form(s), recruitment materials, and all participant materials will be submitted to the IRB for review and approval. Approval of both the protocol and the consent form(s) is obtained before any participant is consented. Any amendment to the protocol will require review and approval by the IRB before the changes are implemented to the study. All changes to the consent form(s) will be IRB approved; a determination will be made regarding whether a new consent needs to be obtained from participants who provided consent, using a previously approved consent form. Authorized representatives of the sponsor, study monitors, representatives of the Institutional Review Board (IRB), or regulatory agencies may inspect all documents and records required to be maintained by the investigator. The clinical study site will permit access to such records. Adverse events (AEs) are reported to the NCH IRB at least annually. AEs are reported to the DSMB at each meeting. AEs are reported to NCI according to their recommended timelines. Per NCH HRP-103 (updated 12/10/18), SAEs determined to be related to the intervention and unexpected are reported to the NCH IRB within 5 business days of discovery; other SAEs are reported to the NCH IRB annually. *S*AEs are reported to the DSMB and to NCI according to their recommended timelines.
Protocol amendments	25	The study team will review protocol deviations on an ongoing basis and will implement corrective actions when the quantity or nature of deviations are deemed to be at a level of concern. This protocol defines a protocol deviation as any noncompliance with the clinical trial protocol, International Council on Harmonization Good Clinical Practice (ICH GCP), or Manual of Procedures (MOP) requirements. The noncompliance may be either on the part of the participant, the investigator, or the study site staff. As a result of deviations, corrective actions will be developed by the site and implemented promptly, if needed.

Consent or assent	26a	Consent forms describing in detail the study intervention, study procedures, and risks are given to the participant and written documentation of informed consent is required prior to starting any data collection procedures or randomizing the family. Families will be given a copy of the signed consent form for their records.<br><br>Caregivers will provide informed consent for their children aged 12-17 years, as well as themselves. Child participants who are 18 years old or older will provide consent for themselves. Children, aged 12-17, will additionally provide assent. Informed consent and assent (if applicable) will be obtained, prior to any study procedures taking place. During the process, the study will be described to participants and families will have the opportunity to discuss participation, before enrolling. Throughout the study, participants will be reminded of the voluntary nature of their involvement.<br><br>Consent will take place either in-person or via remote connection. In-person participants will each receive a copy of the consent form to follow along as the study staff reviews its content. The study staff will then collect all participants' signatures on a single form. Consent will be obtained from remote participant(s) by sending an electronic REDCap link via email. During the process, participants will be connected in via phone or Webex and will have the opportunity to discuss participation. Participants will be provided with an electronic copy of the consent form after submitting the REDCap version.<br><br>Trained study staff will obtain consent according to IRB policy, SOP: Informed Consent Process for Research (HRP-090) to ensure participants understand and do not feel coerced into consenting.
	26b	NA

Confidentiality	27	Data that could be used to identify a specific study participant will be held in strict confidence within the research team. No personally-identifiable information from the study will be released to any unauthorized third party without prior written approval of the funding agency. All research activities will be conducted in as private a setting, as much as possible.<br><br>Authorized representatives of the sponsor, study monitors, representatives of the Institutional Review Board (IRB), or regulatory agencies may inspect all documents and records required to be maintained by the investigator. The clinical study site will permit access to such records.<br><br>The study participant's contact information will be securely stored for internal use during the study. At the end of the study, all records will continue to be kept in a secure location for as long a period as dictated by the reviewing IRB, Institutional policies, or other requirements.<br><br>Study participant research data, which is for purposes of statistical analysis and scientific reporting, will be collected and maintained in REDCap, a secure electronic data capture system on the NCH research internet server. Individual participants and their research data will be identified by a unique study identification number. Other types of data (e.g., audio recordings from the interviews and intervention, paper surveys) may be kept in a locked cabinet within a locked office at the hospital or on an NCH computer. Interviews will be audio recorded and transcribed verbatim. Both the audio files and transcriptions will be stored on a secured intranet server only accessible by research staff. All identifying information will be removed from the transcripts and audio files will be deleted after data collection is completed. Only direct study personnel will have access to this information. The study data entry, study management systems, and related study files will be secured and password protected.
Declaration of interests	28	The principal investigator and other study staff have no financial and or other competing interests for the overall trial to disclose.

Access to data	29	It is NIH policy that the results and accomplishments of the activities that it funds should be made available to the public (see https://grants.nih.gov/policy/sharing.htm). If data are shared, the PI will ensure all mechanisms used to share data will include proper plans and safeguards for the protection of privacy, confidentiality, and security for data dissemination and reuse (e.g., all data will be thoroughly de-identified and will not be traceable to a specific study participant). Plans for archiving and long-term preservation of the data will be implemented, as appropriate. In addition, this study will be conducted in accordance with the following publication and data sharing policies and regulations:<br>• National Institutes of Health (NIH) Public Access Policy, which ensures that the public has access to the published results of NIH funded research. It requires scientists to submit final peer-reviewed journal manuscripts that arise from NIH funds to the digital archive PubMed Central upon acceptance for publication.<br>• This study will comply with the NIH Data Sharing Policy and Policy on the Dissemination of NIH-Funded Clinical Trial Information and the Clinical Trials Registration and Results Information Submission rule. As such, this trial will be registered at ClinicalTrials.gov, and results information from this trial will be submitted to ClinicalTrials.gov. In addition, every attempt will be made to publish results in peer-reviewed journals.
Ancillary and post-trial care	30	NA
Dissemination policy	31a	Results of the study will be submitted to ClinicalTrials.gov no later than one year after the final study visit. Information submitted will include participant flow information, baseline characteristics of the enrolled participants, results for primary and secondary outcomes (fertility preservation uptake, decision quality), and adverse events. Results of the study will also be disseminated via conference presentations (see Career Development Plan) and manuscripts in academic journals.
	31b	Data from the RCT will be presented at scientific conferences (such as the Oncofertility Consortium) and published in peer-reviewed journals. The PI and study team will be responsible for dissemination.
	31c	The Principal Investigator, Dr. Nahata will ensure that this clinical trial will be registered in ClinicalTrials.gov with descriptive information, recruitment information, location and contact information, and administrative information no later than 21 calendar days after enrollment of the first participant.
**Appendices**		
Informed consent materials	32	See Appendix A

Biological specimens	33	NA

*It is strongly recommended that this checklist be read in conjunction with the SPIRIT 2013 Explanation & Elaboration for important clarification on the items. Amendments to the protocol should be tracked and dated. The SPIRIT checklist is copyrighted by the SPIRIT Group under the Creative Commons "Attribution-NonCommercial-NoDerivs 3.0 Unported" license.

References

1. Ries L, Melbert D, Krapcho M, et al. SEER cancer statistics review, 1975–2005. *Bethesda, MD: National Cancer Institute.* 2008;2999.
2. Axtell L, Asire A, Myers M. Cancer patient survival report No. 5. *Bethesda, MD: National Institute of Health.* 1976.
3. Hudson MM, Mertens AC, Yasui Y, et al. Health status of adult long-term survivors of childhood cancer: a report from the Childhood Cancer Survivor Study. *Jama.* 2003;290(12):1583-1592.
4. Green DM, Liu W, Kutteh WH, et al. Cumulative alkylating agent exposure and semen parameters in adult survivors of childhood cancer: a report from the St Jude Lifetime Cohort Study. *The Lancet Oncology.* 2014;15(11):1215-1223.
5. Brignardello E, Felicetti F, Castiglione A, et al. Endocrine health conditions in adult survivors of childhood cancer: the need for specialized adult-focused follow-up clinics. *European journal of endocrinology / European Federation of Endocrine Societies.* 2013;168(3):465-472.
6. Kenney LB, Laufer MR, Grant FD, Grier H, Diller L. High risk of infertility and long term gonadal damage in males treated with high dose cyclophosphamide for sarcoma during childhood. *Cancer.* 2001;91(3):613-621.
7. Kenney LB, Duffey-Lind E, Ebb D, Sklar CA, Grier H, Diller L. Impaired testicular function after an ifosfamide-containing regimen for pediatric osteosarcoma: a case series and review of the literature. *Journal of pediatric hematology/oncology.* 2014;36(3):237-240.
8. Stein DM, Victorson DE, Choy JT, et al. Fertility Preservation Preferences and Perspectives Among Adult Male Survivors of Pediatric Cancer and Their Parents. *Journal of adolescent and young adult oncology.* 2014;3(2):75-82.
9. Nilsson J, Jervaeus A, Lampic C, et al. 'Will I be able to have a baby?' Results from online focus group discussions with childhood cancer survivors in Sweden. *Hum Reprod.* 2014;29(12):2704-2711.
10. Ellis SJ, Wakefield CE, McLoone JK, Robertson EG, Cohn RJ. Fertility concerns among child and adolescent cancer survivors and their parents: A qualitative analysis. *Journal of psychosocial oncology.* 2016;34(5):347-362.
11. Benedict C, Shuk E, Ford JS. Fertility Issues in Adolescent and Young Adult Cancer Survivors. *Journal of adolescent and young adult oncology.* 2016;5(1):48-57.
12. Armuand GM, Wettergren L, Rodriguez-Wallberg KA, Lampic C. Desire for children, difficulties achieving a pregnancy, and infertility distress 3 to 7 years after cancer diagnosis. *Supportive care in cancer : official journal of the Multinational Association of Supportive Care in Cancer.* 2014;22(10):2805-2812.
13. Lehmann V, Keim MC, Nahata L, et al. Fertility-related knowledge and reproductive goals in childhood cancer survivors: short communication. *Hum Reprod.* 2017;32(11):2250-2253.
14. Klosky JL, Simmons JL, Russell KM, et al. Fertility as a priority among at-risk adolescent males newly diagnosed with cancer and their parents. *Supportive care in*

cancer : official journal of the Multinational Association of Supportive Care in Cancer. 2015;23(2):333-341.
15. Nahata L, Caltabellotta NM, Yeager ND, et al. Fertility perspectives and priorities among male adolescents and young adults in cancer survivorship. *Pediatric blood & cancer.* 2018;65(7):e27019.
16. Chong AL, Gupta A, Punnett A, Nathan PC. A cross Canada survey of sperm banking practices in pediatric oncology centers. *Pediatr Blood Cancer.* 2010;55(7):1356-1361.
17. Schover LR, Brey K, Lichtin A, Lipshultz LI, Jeha S. Knowledge and experience regarding cancer, infertility, and sperm banking in younger male survivors. *Journal of clinical oncology : official journal of the American Society of Clinical Oncology.* 2002;20(7):1880-1889.
18. Bann CM, Treiman K, Squiers L, et al. Cancer Survivors' Use of Fertility Preservation. *Journal of women's health.* 2015;24(12):1030-1037.
19. Grover NS, Deal AM, Wood WA, Mersereau JE. Young Men With Cancer Experience Low Referral Rates for Fertility Counseling and Sperm Banking. *Journal of oncology practice.* 2016;12(5):465-471.
20. Nahata L, Cohen LE, Yu RN. Barriers to fertility preservation in male adolescents with cancer: it's time for a multidisciplinary approach that includes urologists. *Urology.* 2012;79(6):1206-1209.
21. Flink DM, Sheeder J, Kondapalli LA. A Review of the Oncology Patient's Challenges for Utilizing Fertility Preservation Services. *Journal of adolescent and young adult oncology.* 2016.
22. Panagiotopoulou N, van Delft FW, Hale JP, Stewart JA. Fertility Preservation Care for Children and Adolescents with Cancer: An Inquiry to Quantify Professionals' Barriers. *Journal of adolescent and young adult oncology.* 2017;6(3):422-428.
23. Campbell JE, Assanasen C, Robinson RD, Knudtson JF. Fertility Preservation Counseling for Pediatric and Adolescent Cancer Patients. *Journal of adolescent and young adult oncology.* 2016;5(1):58-63.
24. Galvin KM, Clayman ML. Whose future is it? Ethical family decision making about daughters’ treatment in the oncofertility context. *Oncofertility.* 2010:429-445.
25. Ellis SJ, Wakefield CE, McLoone JK, Robertson EG, Cohn RJ. Fertility concerns among child and adolescent cancer survivors and their parents: a qualitative analysis. *Journal of psychosocial oncology.* 2016;34(5):347-362.
26. Nahata L, Gerhardt CA, Quinn GP. Fertility Preservation Discussions With Male Adolescents With Cancer and Their Parents:“Ultimately, It’s His Decision”. *JAMA pediatrics.* 2018;172(9):799-800.
27. Nurmi J-E, Poole ME, Kalakoski V. Age differences in adolescent future-oriented goals, concerns, and related temporal extension in different sociocultural contexts. *Journal of Youth and Adolescence.* 1994;23(4):471-487.
28. Flynn JS, Russell KM, Lehmann V, Schenck LAM, Klosky JL. Parent recommendation to bank sperm among at-risk adolescent and young adult males with cancer. *Pediatric Blood & Cancer.* 2020;67(10):e28217.
29. Klosky JL, Flynn JS, Lehmann V, et al. Parental influences on sperm banking attempts among adolescent males newly diagnosed with cancer. *Fertility and sterility.* 2017;108(6):1043-1049.
30. Klosky JL, Wang F, Russell KM, et al. Prevalence and predictors of sperm banking in adolescents newly diagnosed with cancer: examination of adolescent, parent, and provider factors influencing fertility preservation outcomes. *Journal of clinical oncology.* 2017;35(34):3830.
31. Nahata L, Olsavsky A, Dattilo TM, et al. Parent–Adolescent Concordance Regarding Fertility Perspectives and Sperm Banking Attempts in Adolescent Males With Cancer. *Journal of Pediatric Psychology.* 2021;46(10):1149-1158.
32. Theroux CI, Hill KN, Olsavsky AL, et al. Satisfaction with Fertility Preservation Decisions among Adolescent Males with Cancer: A Mixed Methods Study. *Cancers.* 2021;13(14):3559.
33. Elwyn G, Dehlendorf C, Epstein RM, Marrin K, White J, Frosch DL. Shared decision making and motivational interviewing: achieving patient-centered care across the spectrum of health care problems. *The Annals of Family Medicine.* 2014;12(3):270-275.

34. Emmons KM, Rollnick S. Motivational interviewing in health care settings: opportunities and limitations. *American journal of preventive medicine.* 2001;20(1):68-74.
35. International Q. 2021.
36. Glaser BG. The constant comparative method of qualitative analysis. *Social problems.* 1965;12(4):436-445.

Appendix A

CONSENT TO PARTICIPATE IN A CLINICAL RESEARCH STUDY

STUDY TITLE: Fertility Preservation Discussions And Decisions (FP – DAD)

PRINCIPAL INVESTIGATOR: Leena Nahata, MD

CONTACT TELEPHONE NUMBER: (614) 722-2828 (9am-5pm, Monday-Friday)

STUDY SPONSOR: National Institute of Health (NIH)

PARTICIPANT'S NAME: ________________________ DATE OF BIRTH: _________________

NOTE: The words "you" and "your" are used in this consent form. These words refer to the study volunteer whether a child or an adult.

Key Information About This Study

The following is a short summary of this study to help you decide whether or not to participate. More detailed information follows later in this form.

Very little is known about how we can help adolescent and young adults (AYAs) and their caregivers make decisions about fertility preservation (sperm banking) before beginning cancer treatment. The purpose of this study is to see if having a guided conversation about fertility preservation increases preservation rates and/or satisfaction with the decision among AYA males with cancer.

Study participation: Everyone will be asked to complete questionnaires. Families will then be randomized to either standard care or a brief guided conversation about fertility preservation. Half of AYAs and caregivers will be asked to complete a questionnaire about demographic information only. The other half will be asked to complete questionnaires about demographics and about fertility preservation and participate in a discussion about those questions with a trained interventionist. Both groups will be asked to complete additional questionnaires and participate in a brief, audio-recorded interview about 1 month from now and again at 1 year.

Study visits: You will be asked to take part in 3 study visits. The first one will happen today. The second one will happen about month from now. The third one will happen about a year from now. Each one will take about 30 minutes. See a more detailed discussion later in this form.

The main risk of the study is that you could feel irritated or upset when answering questions, but it may be more likely that you find them a little boring. Other risks are listed later in this

form.
Although there may be no benefit to you from being in this study, we hope to learn something that could help others.

If you are interested in learning more about this study, please continue reading below.

1) INTRODUCTION

We invite you to be in this research study. Using this form as a guide, we will explain the study to you. If you have any questions about the study, please ask. By signing this form, you agree to be in this study. If you do not want to be in this study, all regular and standard medical care will still be available to you here at Nationwide Children's Hospital. Participation is voluntary. You can leave this study at any time.

You will be given a signed and dated copy of the consent and the assent forms.

2) WHERE WILL THE STUDY BE DONE AND HOW MANY SUBJECTS WILL TAKE PART?

This study will be done at Nationwide Children's Hospital. We hope to enroll 40 families of AYAs recently diagnosed with cancer.

3) WHAT WILL HAPPEN DURING THE STUDY AND HOW LONG WILL IT LAST?

To determine whether our survey questions and discussion help increase fertility preservation/sperm banking rates, we need two groups of AYA males with cancer. Both groups will be asked to answer demographic questions. One group will also be asked to answer questions about their (or their son's) goals and feelings about parenthood and sperm banking. Upon completion of these questions, a summary will be generated of responses to these questions. Then, a trained interventionist will review responses in front of all participating family members. This means that participating family members and our trained interventionist will see family members' responses. The trained interventionist will guide a discussion amongst participating family members about responses to these questions.

To determine which group you are in, families will be randomized to one of two groups. Randomized means that each participant will be picked by chance, like flipping a coin or drawing straws to get either the demographic questions and standard care or the demographic and fertility preservation questions plus the guided discussion. Each participant has a 1 in 2 chance of being assigned to the guided discussion.

This study will last about 1 year. It will include study activities now, in about 1 month, and about 1 year from now. Each of these meetings will last about 30 minutes. These activities we do at these meetings include answering survey questions, having a guided discussion, and completing a brief audio-recorded interview.

Visit 1:
Everyone will be asked to complete a demographic questionnaire.

Patients and caregivers who are randomized to the guided discussion group will also be asked to complete an additional questionnaire regarding goals and feelings about parenthood and sperm banking. Caregivers will be asked to complete questionnaires about both their own and their son's goals and feelings. This questionnaire should take about 10 minutes for patients and caregivers to complete. The guided discussion should take about 20 minutes.

In addition, study staff will review medical records to obtain information about your diagnosis, treatment, and sperm banking decision. This information will be used in the study to examine the importance of medical factors in understanding decision making and satisfaction.

Visits 2 and 3: After about one month and after about one year, study staff will contact you to complete another questionnaire about family communication and how you feel about your decision. You will also be asked to complete a brief audio recorded interview. The interview will ask more about your decision about fertility preservation, how you feel about that decision, and how being in this study has impacted you. The questionnaire and interview should take about 30 minutes to complete.

4) <u>WHAT ARE THE RISKS OF BEING IN THIS STUDY?</u>

We believe that there is very little chance that bad things will happen as a result of being in this study. It is possible that you could feel irritated or upset when answering questions, but it may be more likely that you find them a little boring. If you do find any of the questions upsetting or don't want to answer a question, you don't have to, and the study staff will be available to discuss this with you further. You can also take breaks while completing the questionnaires. There may be other risks that are not known at this time.

Although we will take every precaution, there is a small chance of loss of confidentiality of your study information.

5) <u>ARE THERE BENEFITS TO TAKING PART IN THIS STUDY</u>?

Although there may be no benefit to you from being in this study, we hope to learn something that could help others.

6) <u>WHAT ARE THE COSTS AND REIMBURSEMENTS</u>?

It will not cost you anything to participate in this study. For your time and inconvenience, each person will receive a $5 meal card after their first visit. You will receive $20 for the second visit, and $30 for the third visit paid on a debit card designed for clinical research. When a study visit is completed, funds will be approved and automatically loaded onto your card. If the card is lost or stolen, please call the study coordinator for a replacement card.

If you receive $600 or more in a calendar year from participating in research studies, you will be issued a 1099 IRS Form to file with your income taxes.

7) <u>WHAT HAPPENS IF BEING IN THIS STUDY CAUSES INJURIES?</u>

We believe that there is very little chance that injuries will happen as a result of being in this study.

8) WHAT HAPPENS IF I DO NOT FINISH THIS STUDY?

It is your choice to be in this study. You may decide to stop being in this study at any time. If you decide to stop being in this study, call the study team at the number on page 1 of this form to see if there are any medical issues about stopping. If you stop being in the study, there will be no penalty or loss of benefits to which you are otherwise entitled.

If at any time the Principal Investigator believes that this study is not good for you, the study team will contact you about stopping. If the study instructions are not followed, participation in the study may also be stopped. If unexpected medical problems come up, the Principal Investigator may decide to stop your participation in the study.

9) OTHER IMPORTANT INFORMATION

If you are an employee of Nationwide Children's Hospital or the Research Institute at Nationwide Children's Hospital, your job or performance review will not be affected in any way if you decline to participate or withdraw your consent to participate in this study.

A description of this clinical trial will be available on http://www.ClinicalTrials.gov, as required by U.S. Law. This website will not include information that can identify you. At most, the website will include a summary of the results. You can search this website at any time.

The final study results will not be shared with you individually. However, at some time, a final study summary will be available on the ClinicalTrials.Gov (http://clinicaltrials.gov) website.

The Principal Investigator is an employee of The Research Institute at Nationwide Children's Hospital and The Ohio State University and is being paid for her time and knowledge needed to do this study.

Nationwide Children's Hospital is a teaching hospital and we are committed to doing research. Doing research will enable us to learn and provide the best care for our patients and families. You may be asked to participate in other research studies in the future. You have the right to decide to participate or decline to participate in any future studies. We will not share your contact information with researchers outside Nationwide Children's Hospital.

10) HOW WILL MY STUDY INFORMATION BE KEPT PRIVATE?

This research is covered by a Certificate of Confidentiality from the National Institutes of Health. This means that the researchers cannot release or use information, documents, or samples that may identify you in any action or suit unless you say it is okay. They also cannot provide them as evidence unless you have agreed. This protection includes federal, state, or local civil, criminal, administrative, legislative, or other proceedings. An example would be a court subpoena.

There are some important things that you need to know. The Certificate DOES NOT stop reporting that federal, state or local laws require. Some examples are laws that require reporting of child or elder abuse, some communicable diseases, and threats to harm yourself or others. The Certificate CANNOT BE USED to stop a sponsoring United States

federal or state government agency from checking records or evaluating programs. The Certificate DOES NOT stop disclosures required by the federal Food and Drug Administration (FDA). The Certificate also DOES NOT prevent your information from being used for other research if allowed by federal regulations.

Researchers may release information about you when you say it is okay. For example, you may give them permission to release information to insurers, medical providers or any other persons not connected with the research. The Certificate of Confidentiality does not stop you from willingly releasing information about your involvement in this research. It also does not prevent you from having access to your own information.

Information collected for this study includes information that can identify you. This is called "protected health information" or PHI. By agreeing to be in this study, you are giving permission to this study team to collect, use, and share your PHI for this research study. Information collected is the property of Nationwide Children's Hospital, its affiliated entities, and/or the sponsor.

Some of the information collected as part of this study will be sensitive, such as information relating to your cancer treatment.

PHI that may be used or shared will include: complete address, telephone number, dates (treatment dates, birth date), email address, medical record number, and voice recordings.

People or Companies authorized to use, share, and receive PHI collected or created by this research study:

- PI and study staff
- The Nationwide Children's Hospital Institutional Review Board (the committee that reviews all human subject research)
- Nationwide Children's Hospital internal auditors
- National Institute of Health (NIH)
- The Office for Human Research Protections (OHRP) (the federal government office that oversees human subject research)

Because of the need to give information to these people, absolute confidentiality cannot be guaranteed. Information given to these people may be further shared by them and no longer be protected by federal privacy rules.

Reason(s) why the use or disclosure is being made: We will record and use/share your PHI to keep track of participants in the research and contact you in the future about progress of the study and other possible chances for involvement in research. This information will also be used to describe the diagnostic and treatment characteristics of the group of participants and evaluate whether outcomes vary for kids with different levels of medical risk.

You may decide not to authorize the use and disclosure of your PHI. However, if it is needed for this study, you will not be able to be in this study. If you agree to be in this study and later decide to withdraw your participation, you may withdraw your authorization to use your PHI. This request must be made in writing to the Principal Investigator at Nationwide Children's

Hospital, 700 Children's Drive, Columbus, OH 43205. If you withdraw your authorization, no new PHI may be collected and the PHI already collected may not be used unless it has already been used or is needed to complete the study analysis and reports.

PHI will only be shared with the groups listed above, but if you have a bad outcome or adverse event from being in this study, the study team or other health care providers may need to look at your entire medical records.

The results from this study may be published but your identity will not be revealed.

The PHI collected or created under this research study will be used or shared as needed until the end of the study. The records of this study will be kept for an indefinite period of time and your authorization to use or share your PHI will not expire.

11) <u>WHOM SHOULD I CALL IF I HAVE QUESTIONS OR PROBLEMS?</u>

If you have questions about anything while on this study or you have been injured by the research, you may contact the Principal Investigator at (614) 722-2828, Monday – Friday, between 9-5 pm.

If you have questions, concerns, or complaints about the research; if you have questions about your rights as a research volunteer; if you cannot reach the Principal Investigator; or if you want to call someone else, call (614) 722-2708, Nationwide Children's Hospital Institutional Review Board, (the committee that reviews all research involving human subjects at Nationwide Children's Hospital).

Signature Block for Children Participant ☐ **N/A, Adult**

Your signature documents your permission for the named child to take part in this research.

____

Printed name of child

____ _________________________

Signature of parent or individual legally authorized to consent Date & Time AM/PM
to the child’s general medical care

____

Printed name of parent or individual legally authorized to consent
to the child’s general medical care

____

Relationship to Participant

Note: Investigators are to ensure that individuals who are not parents can demonstrate their legal authority to consent to the child’s general medical care. Contact Legal Services if any questions arise.

____ _________________________

Signature of second parent or individual legally authorized to Date & Time AM/PM
consent to the child’s general medical care

____

Printed name of second parent or individual legally authorized to
consent to the child’s general medical care

____

Relationship to Participant

If signature of second parent not obtained, indicate why: (select one)

- Not required by IRB
- Second parent is deceased
- Second parent is unknown
- Second parent is incompetent
- Second parent is not reasonably available
- Only one parent has legal responsibility for the care and custody of the child

Signature of person obtaining consent Date & Time AM/PM

Printed name of person obtaining consent

Assent

Signature of subject Date & Time AM/PM

❑ Not obtained because the capability of the subject is so limited that the subject cannot reasonably be consulted.

☐ **N/A, Witness not required**

My signature below documents that the information in the consent document and any other written information was accurately explained to, and apparently understood by, the subject, and that consent was freely given by the subject.

Signature of witness to consent process Date & Time AM/PM

Printed name of person witnessing consent process

Signature Block for Adult Participation ☐ **N/A, Pediatric Participant**

Your signature documents your permission to take part in this research.

____

Signature of subject Date & Time AM/PM

____

Printed name of subject

____

Signature of person obtaining consent Date & Time AM/PM

____

Printed name of person obtaining consent

☐ **N/A, Witness not required**

My signature below documents that the information in the consent document and any other written information was accurately explained to, and apparently understood by, the subject, and that consent was freely given by the subject.

____

Signature of witness to consent process Date & Time AM/PM

____

Printed name of person witnessing consent process

Signature Block for Adult Participation ☐ **N/A, Pediatric Participant**

Your signature documents your permission to take part in this research.

Signature of subject Date & Time AM/PM

Printed name of subject

Signature of person obtaining consent Date & Time AM/PM

Printed name of person obtaining consent

☐ **N/A, Witness not required**

My signature below documents that the information in the consent document and any other written information was accurately explained to, and apparently understood by, the subject, and that consent was freely given by the subject.

Signature of witness to consent process Date & Time AM/PM

Printed name of person witnessing consent process

Signature Block for Adult Participation ☐ **N/A, Pediatric Participant**

Your signature documents your permission to take part in this research.

____

Signature of subject Date & Time AM/PM

____

Printed name of subject

____

Signature of person obtaining consent Date & Time AM/PM

____

Printed name of person obtaining consent

☐ **N/A, Witness not required**

My signature below documents that the information in the consent document and any other written information was accurately explained to, and apparently understood by, the subject, and that consent was freely given by the subject.

____

Signature of witness to consent process Date & Time AM/PM

____

Printed name of person witnessing consent process
