# Supplementary material for: Study protocol for fertility preservation discussions and decisions: A family-centered psychoeducational intervention for male adolescents and emerging adults newly diagnosed with cancer and their families
Source: PLoS One. 2022 Feb 16;17(2):e0263886. doi: 10.1371/journal.pone.0263886 (PMC8849538; doi:10.1371/journal.pone.0263886)
Supplement: S2 File — (DOCX) [file pone.0263886.s002.docx]

**PROTOCOL TITLE:**

Fertility Preservation Discussions And Decisions (FP – DAD)

**PRINCIPAL INVESTIGATOR:**

Name: Dr. Leena Nahata

Department/Center: Center for Biobehavioral Health, Nationwide Children’s Hospital

Telephone Number: 1 614 722 4425

Email Address: [Leena.Nahata@nationwidechildrens.org](mailto:Leena.Nahata@nationwidechildrens.org)

**VERSION NUMBER/DATE:**

V6, 25 August 2021

**REVISION HISTORY**

| **Revision #** | **Version # and Date** | **Summary of Changes** | **Consent Change?** |
| --- | --- | --- | --- |
| 1 | 2.0 09/28/2020 | We updated information on the current status of our study, outlined procedures for connecting a remote participant in, and updated the description of our surveys/methods to reflect changes made to those areas. We also changed the name of the study. | Yes |
| 2 | 3.0 12/15/2020 | We updated information to include procedures for connecting an entire family in via Webex | No |
| 3 | 4.0  01/08/2021 | We included an additional questionnaire to give to families during visit 2. | No |
| 4 | 5.0 06/03/2021 | We updated our remote consent processes to include REDCap. | No |
| 5 | 6.0 | We included questionnaires to give to families during visit 3. | No |

# Statement of Compliance

The trial will be carried out in accordance with International Council on Harmonization Good Clinical Practice (ICH GCP) and the following:

- - United States (US) Code of Federal Regulations (CFR) applicable to clinical studies (45 CFR Part 46, 21 CFR Part 50, 21 CFR Part 56, 21 CFR Part 312, and/or 21 CFR Part 812).

Additionally, National Institutes of Health (NIH)-funded investigators and clinical trial site staff who are responsible for the conduct, management, or oversight of NIH-funded clinical trials have completed Human Subjects Protection and ICH GCP Training.

The protocol, informed consent form(s), recruitment materials, and all participant materials will be submitted to the IRB for review and approval. Approval of both the protocol and the consent form(s) is obtained before any participant is consented. Any amendment to the protocol will require review and approval by the IRB before the changes are implemented to the study. All changes to the consent form(s) will be IRB approved; a determination will be made regarding whether a new consent needs to be obtained from participants who provided consent, using a previously approved consent form.

# Investigator Signature

The signature below constitutes the approval of this protocol and provides the necessary assurances that this study will be conducted according to all stipulations of the protocol, including all statements regarding confidentiality, and according to local legal and regulatory requirements and applicable US federal regulations and ICH guidelines, as described in the *Statement of Compliance* above.

**Principal Investigator:**

| Signed: |  | Date: |  |  |
| --- | --- | --- | --- | --- |
| Name: | Leena Nahata, MD | | |  |
| Titles: | Associate Professor of Clinical Pediatrics, Pediatric Endocrinology  Principal Investigator, Center for Biobehavioral Health | | |  |
| Affiliation: | The Ohio State University and Abigail Wexner Research Institute at Nationwide Children's Hospital | | | |
| Address: | 700 Children's Drive  Columbus, OH 43205 | | | |
| Telephone: | 614-722-4425 | | | |
| Email: | Leena.Nahata@nationwidechildrens.org | | | |

Table of Contents

[Statement of Compliance 2](#_Toc27556621)

[Investigator Signature 2](#_Toc27556622)

[1.0 Study Summary 4](#_Toc27556623)

[2.0 Objectives 4](#_Toc27556624)

[3.0 Background 5](#_Toc27556625)

[4.0 Study Endpoints 6](#_Toc27556626)

[5.0 Study Intervention 7](#_Toc27556627)

[6.0 Procedures Involved* 7](#_Toc27556628)

[7.0 Data and Specimen Banking* 10](#_Toc27556629)

[8.0 Sharing of Results with Subjects* 11](#_Toc27556630)

[9.0 Study Timelines* 11](#_Toc27556631)

[10.0 Inclusion and Exclusion Criteria* 11](#_Toc27556632)

[11.0 Vulnerable Populations* 12](#_Toc27556633)

[12.0 Local Number of Subjects 12](#_Toc27556634)

[13.0 Recruitment Methods 12](#_Toc27556635)

[14.0 Withdrawal of Subjects* 12](#_Toc27556636)

[15.0 Risks to Subjects* 12](#_Toc27556637)

[16.0 Potential Benefits to Subjects* 13](#_Toc27556638)

[17.0 Data Management* and Confidentiality 13](#_Toc27556639)

[18.0 Provisions to Monitor the Data to Ensure the Safety of Subjects* 15](#_Toc27556640)

[19.0 Adverse Events & Serious Adverse Events 16](#_Toc27556641)

[20.0 Unanticipated Problems 17](#_Toc27556642)

[21.0 Provisions to Protect the Privacy Interests of Subjects 17](#_Toc27556643)

[22.0 Compensation for Research-Related Injury 18](#_Toc27556644)

[23.0 Economic Burden to Subjects 18](#_Toc27556645)

[24.0 Consent Process 18](#_Toc27556646)

[25.0 Process to Document Consent in Writing 19](#_Toc27556647)

[26.0 Setting 19](#_Toc27556648)

[27.0 Resources Available 19](#_Toc27556649)

[28.0 Multi-Site Research* 20](#_Toc27556650)

[29.0 Protected Health Information Recording 20](#_Toc27556651)

[30.0 Confidential Health Information 22](#_Toc27556654)

[31.0 References 23](#_Toc27556659)

# Study Summary

| **Study Title** | Fertility Preservation Discussions and Decisions (FP-DAD) |
| --- | --- |
| **Grant Number** | K08CA237338, National Cancer Institute |
| **Study Design** | Randomized Clinical Trial |
| **Primary Objective** | To examine feasibility, acceptability, and preliminary efficacy of a family-centered psychoeducational intervention designed to improve fertility preservation (FP) uptake. |
| **Secondary Objective(s)** | To examine feasibility, acceptability, and preliminary efficacy of a family-centered psychoeducational intervention designed to improve FP decision quality and family communication. |
| **Research Intervention** | A family-centered psychoeducational intervention, which involves a FP decision tool, which is used by a trained interventionist to facilitate communication about FP between patients and caregivers. |
| **IND/IDE #** | NA, Behavioral Intervention |
| **Study Population** | AYA (aged 12-25-years) newly diagnosed with cancer and 70 caregivers will be enrolled in the study. |
| **Sample Size** | 40 |
| **Study Duration for Participants** | 1 year +/- 1 month |
| **Study Specific Abbreviations/ Definitions** | IRB – Institutional Review Board; PI – Primary Investigator; RA – Research Assistant; BTO – Behavioral Trials Office; DSMB – Data Safety Monitoring Board; OHRP – Office of Human Research Protocols; CITI - Collaborative Institutional Training Initiative; QC – Quality Control; eCRF – Electronic Case Report Form; AYA – Adolescent and Young Adult; QoL – Quality of Life; HBM- Health Belief Model; FP – Fertility Preservation; PACS – Parent Adolescent Communication Scale; BSDQ – Brief Subjective Decision Quality |

# Objectives

*Aims:* This randomized clinical trial (RCT) will test the feasibility, acceptability, and preliminary efficacy of a novel family-centered, psychoeducational intervention with known and newly identified factors to improve fertility preservation (FP) uptake, decision quality, and family communication among male adolescents and young adults (AYAs) newly diagnosed with cancer. The primary aim of this project is to examine preliminary efficacy of the psychoeducational intervention with regard to FP uptake.

Hypotheses: The primary hypothesis is that compared to standard of care control group (routine fertility consult at diagnosis, n=20), AYAs in the intervention arm (routine fertility consult at diagnosis + FP Decision Tool and Facilitated Conversation by trained interventionist; n=20) will have higher rates of FP uptake. The secondary hypotheses are that families in the intervention group will report better FP decision quality and report improvements in family communication, compared to those in the control arm.

*Rationale:* Nearly 9,000 males under age 20 are diagnosed with cancer in the U.S. each year^1^. With 5-year survival rates exceeding 80%, the population of male childhood cancer survivors is now close to *200,000^2^*. Unfortunately, approximately 50% of male survivors have impaired fertility^3-5^, which can impede psychosexual development and diminish quality of life (QoL)^6^. Sperm banking is an established and effective method of FP for pubertal males^7^, yet only 25% of eligible adolescents bank sperm prior to treatment^8-12^. As these AYAs complete therapy and approach their reproductive years, many survivors and their parents *regret* missed opportunities for FP and report *distress* about potential infertility^13-16^.

Significant knowledge gaps exist with regard to pediatric FP:

Most research examining FP decisions has focused on females^17,18^, despite a higher prevalence of cancer and treatment-related infertility in males^19^;

Many studies examining FP predictors have been conducted years after therapy and are subject to recall bias^15,20^;

Little attention has been paid to *decision quality* (satisfaction/regret) soon after FP decisions are made;

Although parents make most healthcare decisions even in late adolescence/young adulthood, *family factors* including parent-child communication about FP, have not been examined.

FP interventions have primarily focused on patient-provider communication and increasing knowledge^21-23^, but in a recent study, half of 13-21-year old males who were told they were at risk for infertility still declined FP before treatment^24^. Notably, *parents’ recommendation to bank* was one of the strongest predictors of FP^25,26^. However, research has shown that parents do not prioritize fertility in the setting of their child’s new cancer diagnosis^27,28^. AYA survivors and parents in our recent pilot study had discordant attitudes about FP, with parents significantly underestimating the impact of potential infertility on their sons. Thus, knowledge is critical, but other *individual and family* factors must be identified to explain gaps between those pursue and decline FP. The relative ease but underutilization of sperm banking, as well as the psychosocial implications of infertility, demonstrate a need for novel family-centered interventions at the time of diagnosis to optimize FP uptake and decision quality for this unique and vulnerable population^25,26^. Such interventions have improved parent-child communication about sexual health topics and have shown promise in other conditions such as multiple sclerosis. However, research at the time of diagnosis has been limited by concerns that the burden of participating could be too heavy at such a tumultuous time. Previous research has shown that participating in FP focused research at the time of diagnosis is rated as beneficial or neutral by AYAs and families^29^. In our ongoing observational pilot (IRB17-00883) our recruitment rate is 93% for Visit 1 and 81% for Visit 2, which shows that a study of this kind is feasible at the time of diagnosis.

# Background

A rapidly growing population of male childhood cancer survivors are at risk for infertility and distress. As the number of male cancer survivors rises, it is essential to minimize treatment late effects^2,30^. One of the most prevalent and significant complications among males is infertility^3-5,30,31^, which can impair psychosocial development and reduce quality of life^6^. National guidelines emphasize offering fertility preservation (FP) prior to initiation of cancer therapy^32^, and sperm cryopreservation is an established and generally noninvasive FP method for pubertal males. Early research suggested only males receiving high doses of alkylating agents should bank sperm^33^. However, variable sperm counts following equivalent doses of cyclophosphamide and scenarios in which patients have to move quickly from “low risk” treatments (which transiently impair sperm production) to “high risk” treatments, support the premise that all males receiving chemotherapy and/or gonadal radiation should consider FP at diagnosis^3,5,32,34^. Despite studies showing ~50% of male childhood cancer survivors have fertility impairment^3,4,33,35^, reports from many centers show only ~25% of pubertal males bank sperm prior to treatment^8-12^. As survivors enter their reproductive years, many regret missed opportunities for FP and experience *distress* about potential infertility^11,13-16,36^. Thus, interventions to improve FP uptake would have great potential for reproductive and psychological benefit.


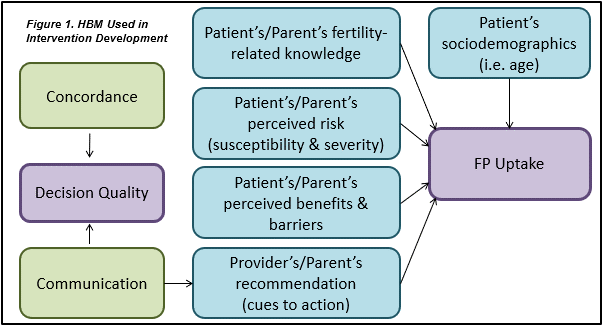
Individual and family factors associated with sperm banking decisions remain poorly understood. Young age, cost, inadequate knowledge, and urgency to start treatment are common barriers to FP among AYA males with newly diagnosed cancer^9,37-40^. As a result, most fertility counseling and FP interventions have targeted healthcare providers and systems or provider-patient interactions^21-23,41^. While these are critical factors, less than half of male AYA advised about FP in a recent study actually banked sperm^24^, indicating knowledge is not sufficient. Other individual and family factors influencing FP decisions must be identified. The Health Belief Model (HBM) is often used to explain or predict an individual’s health-related behavior and uptake of health services^42,43^, and has informed effective interventions^44^. In the HBM (Figure 1), uptake of a procedure (FP) is dependent on the interplay of sociodemographic characteristics, fertility knowledge, perceived benefits and barriers (to FP), perceived risk, and cues to action (provider and parent recommendation). Studies in healthy women at risk for infertility have shown that the HBM effectively predicts FP uptake and engagement in fertility-optimizing behaviors^45,46^. More recently, the HBM has shown applicability to AYA males when FP decisions were studied retrospectively (*after* treatment began); higher perceived benefits of FP were associated with FP attempts^26^. The HBM was the guiding principle in developing the family-centered, psychoeducational decision tool intervention used in this study.

The role of parents/caregivers in FP decisions is also understudied. This is relevant for 4 main reasons:

1. *Parents are the primary medical decision makers for their children into young adulthood*. They act as gatekeepers and often manage what and how their children receive information about their illness and treatment. Research shows parents play an important role in decisions to bank sperm^26,47-49^.
2. *Parents report challenges in how and when to discuss fertility with their children^14,48^*. A recent study found parents of children and AYAs (up to 20 years old) struggled to broach this topic^14^. Sperm banking discussions frequently include sensitive issues, such as sexual milestones including masturbation to collect a semen sample (and use of pornographic material), sexual history/experiences, and acceptability of biological versus alternative methods of parenthood^26,50^. These topics are inherently challenging, and some parents may even oppose healthcare providers initiating discussions due to religious, cultural, or personal views^48,51^.
3. *Many parents poorly estimate their sons/daughters’ reproductive desires and worries, and struggle with separating their own desires from those of their children^52^.* A 2012 study in adolescent females recently diagnosed with cancer showed parents underestimated their daughters’ reproductive concerns^28^. Similarly, AYA males recently diagnosed with cancer ranked fertility in the top three life priorities, whereas parents were more concerned with their sons’ health^27^. Further, while many parents want to be grandparents in the future, they are often more willing to consider alternate means of family building (e.g. adoption) than their sons^53^. Discordance, and/or parents’ lack of awareness of their sons’ reproductive goals and concerns, may ultimately lead to lower decision quality.
4. *Notably,* *a recent study among 13-21-year old males within one week of initiation of cancer treatment demonstrated parents’ recommendation to bank sperm, as compared with the medical team, was the most robust predictor of a FP attempt^26,49^.* Thus, an intervention designed to increase caregiver-child communication should be examined within an established health behavior framework to examine its preliminary efficiency at increasing rates of FP in AYAs.

We completed an observational pilot study (IRB17-00883) testing feasibility and initial effects of a decision tool examining individual and family factors that impact FP decisions. The pilot informed the development of the decision tool that will be used in this study intervention, as well as the interviews used at the 1-month and 1-year follow-up. Recruitment rates were above 90% for visit 1, further demonstrating the feasibility of this type of study at the time of diagnosis.

# Study Endpoints

The overarching goal of this project is to examine preliminary efficacy of the psychoeducational intervention in a pilot RCT. The *primary endpoint* is FP uptake following the intervention. The *secondary endpoints* are the Brief Subjective Decision Quality (BSDQ) questionnaire scores at 1-month and 1-year post-intervention and the change in Parent Adolescent Communication Scale (PACS) scores from baseline to 1-month and 1-year post-intervention.

# Study Intervention

*Intervention Description:* The intervention will be administered by a trained interventionist who has participated in ENRICH/ECHO, an oncofertility communication training program. The interventionist will administer the digital FP Decision Tool to families in the intervention arm and will facilitate a guided discussion about responses and discrepancies. The FP Decision Tool asks 25 questions examining each AYA’s thoughts or feelings regarding parenthood and fertility preservation. The parent version asks the same 25 question, plus an additional 9 questions asking the parent to rate their son’s feelings on certain topics (i.e. whether their son wants to have a child, whether their son would be as happy with an adopted child versus a biological child). Items are coded based on the domains of the HBM (perceived benefits, perceived barriers, perceived threats, self-efficacy, and cues to action). The decision tool was developed using the HBM and is based on factors associated with FP uptake and decision quality in the literature, as well as new factors identified in the preliminary work of this research group.

AYAs and parents independently complete the electronic tool; an automated Quick View report is generated, highlighting misconceptions and parent-AYA discrepancies. The interventionist uses this information to facilitate a conversation with AYA and their caregiver(s). Specifically, the interventionist uses standardized prompts/language to: 1) address knowledge gaps/misconceptions, 2) share participants’ fertility-related values and goals with one another, 3) highlight discrepancies between responses, and 4) promote family communication and consensus building. The facilitated conversation will be audio recorded.

*Intervention Rationale:* The intervention is designed to take participant’s answers and provide a conversation tailored to their questions and needs. It will be carried out by one interventionist that has completed ENRICH/ECHO (oncofertility communication) training to ensure the intervention will not unnecessarily burden patients and families. Additionally, the intervention is designed to be short (less than 20 minutes). It is expected that participants will be present for the entire intervention in order to have their baseline data included in analyses.

*Interventionist Training and Tracking:* The interventionist will have a degree in a health-related field such as psychology or nursing. They will be trained in ENRICH/ECHO, an oncofertility communication training program. This training program include several modules to assist oncology health professionals effectively communicate key information regarding reproductive health to AYA. Additionally, the interventionist will be extensively trained in the use of the FP Decision Tool, the interactive report, and the communication intervention by Drs. Nahata and Gerhardt.

*Intervention Fidelity:* Consistent delivery of the study interventions will be monitored throughout the intervention phase of the study. A random selection of audiotapes of the interactions will be reviewed by trained research staff on an ongoing basis who will complete a checklist of the five points to be covered in the facilitated conversation to monitor intervention fidelity. The five points are: 1) address knowledge gaps/misconceptions by referring families back to the fertility navigator, 2) share participants’ fertility-related values and goals with one another, 3) highlight discrepancies between responses, and 4) promote family communication and consensus building.

# Procedures Involved*

Study Design: This single-site study is an RCT of a family-centered psychoeducational decision tool intervention. Participants will be male AYA (ages 12-25-years) newly diagnosed with cancer and their caregivers. Families will be randomized (1:1) to either receive 1) standard of care fertility consult or 2) standard of care fertility consult + the family-centered psychoeducational decision tool and facilitated communication.

This study seeks to improve FP in AYA males newly diagnosed with cancer by designing and evaluating the feasibility, acceptability, and preliminary efficacy of a family-centered psychoeducational intervention to optimize FP uptake and decision quality. To this effect, we will employ a blinded design. Both control and experimental groups will be pulled from one population (males with new cancer diagnoses) to control for biases and individual differences. Participants will be randomized and staff who consent families and conduct data collection activities will be blind to treatment group. The CBH Behavioral Trials Office (BTO) will maintain the randomization sequence and randomization to study group will be implemented through the data management software REDCap, which allows the randomization sequence generated by the statistician to be allocated via REDCap Randomization Module. In this module, the randomization sequence and group assignments are protected from the view of selected staff who use the REDCap program. Only BTO staff will have REDCap permissions to see the randomization sequence. Only the interventionist will have REDCap permissions to randomize and see the allocated group assignment when they log-on to REDCap. Outcome assessors (RAs) and investigators will be blinded to the intervention and will not have access to treatment allocation information in REDCap.


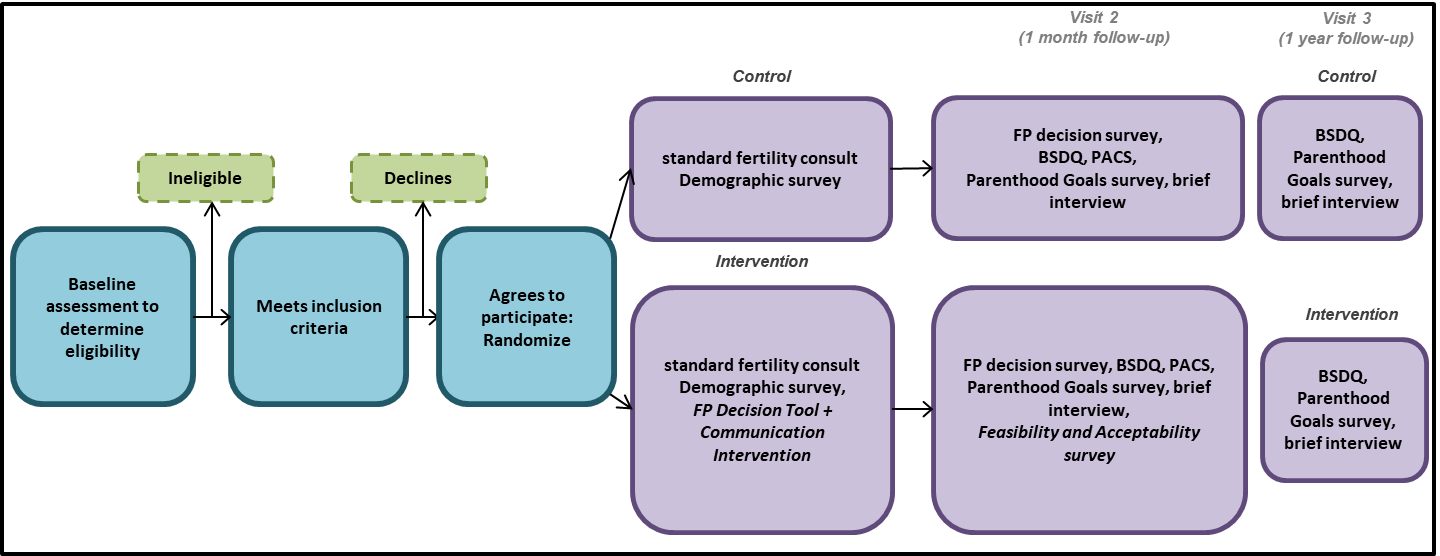


**Baseline (Visit 1):** At baseline (Visit 1), research staff will approach families in-person (at inpatient or outpatient clinic) or via phone, Webex, or email. Families (AYAs and parents) will receive a short briefing on the purpose of the study, provide informed consent, and staff will administer baseline measures after the family has consented to participate in the study. All measures will be completed by the child and both caregivers (when available). If a caregiver is only available remotely, a REDCap link to the survey will be provided electronically, and the caregiver will be included in the intervention remotely (e.g. via phone). If the entire family is only available remotely, participants will be contacted via phone and will be emailed a Webex link and REDCap links to participate in the study. The activities completed at Visit 1 are:

- Informed Consent: Informed consent (and assent for participants 12-17 years of age) is obtained by study staff before data collection procedures or randomization. For adolescents between 12-17 years of age, informed consent will be obtained by a parent or legal guardian, and assent will be provided by the adolescent. Those who are between 18-25-years of age will provide informed consent for themselves to participate.
- Demographics: Research staff ask participants to answer questions about age, race, ethnicity, religion, education, income, whom they live with, and their relationship to their child. AYAs will be asked to answer an additional four questions about their communiation with their parents. These questions were taken from the Parent-Adolescent Communication Scale (PACS).
- Randomization: Families who consent to participate will be randomized (by the interventionist) to the control arm or intervention arm (family-centered psychoeducational intervention) after completing baseline measures. If the family is randomized to the control arm, the interventionist will inform the family that they were randomized to the control group and debrief the family. If the family is randomized to the intervention arm, the interventionist will inform the family that they were randomized to the treatment group and conduct the intervention immediately following randomization.

**1-month Follow-up (Visit 2, Baseline + 1 to 2 months):** At the 1-month follow-up (Visit 2) research staff will contact control and intervention families in-person (at inpatient or outpatient clinic) or via phone or email. Families (AYAs and parents) will be reminded about the study and be asked to complete follow-up activities. All measures will be completed by the child and both caregivers (when available). The activities completed at Visit 2 include:

- FP Decision Survey: Participants will complete a brief survey about their FP decision. Items will include questions such as who participants talked to about their FP decision and who made the final decision regarding FP. Surveys will not be scored but questions will be used to guide analyses.
- Brief Subjective Decision Quality measure (BSDQ): A six-item scale used to measure decision satisfaction and administered to AYA and both parents. Item will be scored into a composite decision satisfaction score (0-7).
- The Parent-Adolescent Communication Scale (PACS): A twenty-item scale used to measure perceived communication between AYA and parents will be administered to AYA and both parents. AYA will rate communication with both mother and father. Parents will only rate communication with son. Item scored into three scales: openness, problems, and a composite communication score.
- Parenthood Goals Survey: A sixteen-item survey used to examine parental and AYA views on parenthood. This survey is a modified version of the FP Decision Tool. This survey will be given to families in both the control and intervention group.
- Feasibility and Acceptability Survey: For the intervention group only, AYA and parents will fill out a twelve-item scale that will be used to assess satisfaction with the intervention structure and content. The scale will include a final open-ended question asking for comments/suggestions about the intervention. The parent survey will include an additional item specific to how the intervention helped them help their child.
- Brief Interview: Study staff will also complete a brief, 15-minute semi-structured interview separately with each participant. The interview will focus on what participants discussed in relation to FP, how they made their decision, how they feel about their decision, and how they feel overall about their fertility counseling experience. The final question will give families the opportunity to share any additional thoughts. Interviews will take place away from other participants to allow each interviewee to speak freely. Interviews will be audio recorded and transcribed verbatim and coded for thematic content by trained research staff.

**1-year Follow-up (Visit 3, Baseline + 1-year (+/- 1 month):** At the 1-year follow-up (Visit 3) research staff will contact control and intervention families in-person (at inpatient or outpatient clinic) or via phone or email. Families (AYAs and parents) will be reminded about the study and research activities. All measures will be completed by the child and both caregivers (when available). The activities completed at Visit 3 include:

- Brief Subjective Decision Quality measure (BSDQ): A seven-item scale used to measure decision satisfaction and administered to AYA and both parents. Item will be scored into a composite decision satisfaction score (0-7).
- Parenthood Goals Survey: A sixteen-item survey used to examine parental and AYA views on parenthood. This survey is a modified version of the FP Decision Tool. This survey will be given to families in both the control and intervention group.
- Brief Interview: Study staff will also complete a brief, 15-minute semi-structured interview separately with each participant. The interview will focus on what participants found helpful in making their decision, how their feelings about their decision have changed over time, what they would have liked to have known prior to their decision, and information and or support related to fertility that will help them moving forward. Interviews will take place away from other participants to allow each interviewee to speak freely. Interviews will be audio recorded and transcribed verbatim and coded for thematic content by trained research staff.

*Risk Minimization:* The benefits of this research outweigh the minimal risk, both for participants and certainly for society, which would benefit substantially from development of feasible interventions to assist in FP decisions for newly diagnosed cancer patients, thus making a favorable risk-benefit ratio. Additionally, previous research has found that participation in family-centered research focused on FP, before treatment begins, is viewed as beneficial or neutral by participants^29^. However, the following procedures are implemented to further minimize risk.

1. Approval of all procedures connected with this study is obtained and maintained from the Institutional Review Board at NCH.
2. Informed consent (and assent for participants 12-17 years of age) will be obtained, prior to any study procedures. Caregivers will provide informed consent for their children aged 12-17 years. Participants over the age of 18 will consent for themselves to participate.
3. Participants will be reminded of the voluntary nature of their involvement.
4. All study personnel who will be working with data or protected health information will be properly trained, which includes completing the online Collaborative Institutional Training Initiative (CITI) certification and undergoing a day-long instructional seminar at the hospital for protecting human subjects and engaging in best practices for responsible conduct of research. Additionally, per NIH policy, clinical trial staff who are involved in the design, conduct, oversight, or management of the trial will be trained in Good Clinical Practice (GCP).
5. Staff will receive extensive training in administration of all questionnaires as well as professional behavior with families. The interventionist will have additional training in ENRICH/ECHO, an oncofertility communication training program.
6. Staff will be trained to contact the PI immediately if an adverse event occurs, and corrective action, including IRB notification, will occur, if necessary.
7. Once enrolled in the study, participants (patients and parent(s)) will receive a study ID number. This number will be entered on their survey, be it paper or online (i.e., using REDCap, an online survey system that is housed securely on the NCH research drive). The only link between the participant’s name and study ID will be an electronic tracking sheet, which will be password protected and stored on the secured research network drive of the hospital. The tracking sheet will contain the following protected health information: name, medical record number, date of birth, address, phone number, email address, diagnosis, and dates of study visits. Only direct study personnel will have access to this file. Participants will be identified in all datasets by their study ID.
8. If participants find any of the questions upsetting, the study team will be available to discuss the concerns or reconnect the participant to their assigned psychosocial provider at Nationwide Children’s Hospital. Participants may also skip any questions that they do not want to answer.
9. Online questionnaire data and voice recordings will be housed behind a secure firewall on the NCH research internet server. Any data that is not collected in a digital format will be kept in a locked cabinet within a locked office at the hospital. Only study personnel will have access to this information.

*Data Collection Procedures:* Data described in Section 6.0 will be collected for all participants. Data will be collected by trained research staff using an online survey tool (e.g., REDCap) or paper and pen questionnaires. Some data may be obtained from the participant’s electronic medical record. Data collected at Visit 1 will be collected before the intervention begins, and data collected at Visits 2 and 3 will be collected after the intervention ends.

*End of Study Definition:* A participant is considered to have completed the study if they completed the baseline assessment, as well as the 1-month and 1-year follow-up assessments.

*Lost-to-Follow-up Definition:* A participant will be considered lost to follow-up if they fail to return for scheduled visits and study staff are unable to contact the participant after at least 3 attempts via phone or email.

# Data and Specimen Banking*

*Data Storage:* Online questionnaire data will be housed behind a secure firewall on the NCH research internet server. Electronic recordings will be stored on NCH computers. Data collected on paper will be entered into electronic study databases and original files will be kept in a locked cabinet within a locked office at the hospital. Only direct study personnel will have access to this information.

*Data Description:* Data described in Section 6.0 will be collected for each participant.

Data Sharing: It is NIH policy that the results and accomplishments of the activities that it funds should be made available to the public (see https://grants.nih.gov/policy/sharing.htm). If data are shared, the PI will ensure all mechanisms used to share data will include proper plans and safeguards for the protection of privacy, confidentiality, and security for data dissemination and reuse (e.g., all data will be thoroughly de-identified and will not be traceable to a specific study participant). Plans for archiving and long-term preservation of the data will be implemented, as appropriate. In addition, this study will be conducted in accordance with the following publication and data sharing policies and regulations:

- National Institutes of Health (NIH) Public Access Policy, which ensures that the public has access to the published results of NIH funded research. It requires scientists to submit final peer-reviewed journal manuscripts that arise from NIH funds to the digital archive PubMed Central upon acceptance for publication.
- This study will comply with the NIH Data Sharing Policy and Policy on the Dissemination of NIH-Funded Clinical Trial Information and the Clinical Trials Registration and Results Information Submission rule. As such, this trial will be registered at ClinicalTrials.gov, and results information from this trial will be submitted to ClinicalTrials.gov. In addition, every attempt will be made to publish results in peer-reviewed journals.

# Sharing of Results with Subjects*

Individual subject results will not be shared directly with participants. Overall study results may be shared with participants via study newsletters or similar.

# Study Timelines*

Participants will be enrolled in this study for approximately 1 year.

We expect it will take 48 months to enroll all participants and that the primary analysis will be completed within one year after enrollment is completed.

# Inclusion and Exclusion Criteria*

*Eligibility:* After an initial and new cancer diagnosis, an automatic referral is placed for a fertility consult and Dr. Nahata is immediately notified of the age and sex of the patient. The patient and family then consult with a fertility navigator to discuss fertility options, per standard of care. During the fertility consult the fertility navigator will assess pubertal status/eligibility for sperm banking, provide verbal and written information about infertility risk and FP options (masturbation/sperm extraction), and then introduce the study. The patient’s eligibility is communicated to study staff. If the participant is eligible, study staff will approach the family for recruitment purposes.

Inclusion Criteria: Child participants must meet all the inclusion criteria to be eligible for the study. The inclusion criteria are:

- Male
- Aged 12-25 years
- Expected to have adjuvant therapy (chemotherapy and/or gonadal radiation) for newly diagnosed cancer
- Pubertal (at least Tanner stage 2-3, eligible for sperm banking as determined in the fertility consult)
- Proficient in English

Primary caregivers of eligible children are also invited to participate if they have participated in the fertility consult. These caregivers may include biological parents, adoptive patterns, other primary caregivers (e.g. grandparents), or same sex parents.

*Exclusion Criteria:* Child participants meeting any of the exclusion criteria at baseline will be excluded from the study. The exclusion criteria are:

- Cognitive deficit that precludes completing measures
- Parents are non-English speaking

# Vulnerable Populations*

This study will include children who have not obtained the legal age to consent. The following safeguards will be followed to protect their rights and welfare: study staff will not approach without at least one parent present, staff will make it clear that if the child feels overwhelmed or uncomfortable they may stop participating at any time, and, when appropriate, staff may approach parents separately from the child to ensure that child is able to complete study measures.

# Local Number of Subjects

This study will enroll 40 male AYAs and 70 caregivers, at the single-site, Nationwide Children’s Hospital. There are approximately 30 males newly diagnosed with cancer in our target age range each year. Taking into account our inclusion/exclusion criteria, infrastructure of the fertility consult service, and recruitment rates in our pilot (>90%), we anticipate completing our recruitment goals within the allocated timeframe.

# Recruitment Methods

At Nationwide Children’s Hospital (NCH), every patient with a new cancer diagnosis has an automatic referral placed to the multidisciplinary Fertility and Reproductive Health Team. The oncofertility navigator meets with the patient and family to assess pubertal status and achievement of sexual milestones (masturbation), review infertility risk, present FP options, and discuss logistics (including cost). After the oncofertility navigator completes the consult and introduces the study to the family, the study team will approach those who are eligible and invite their participation. Study staff will primarily recruit in person, in clinic. As the first assessment will occur immediately after the fertility consult (before cancer treatment), most AYA have two parents present. If participants are unavailable at times of appointments or admissions, study staff may contact families via telephone or email.

If a participant is not present during the time of in person recruitment, but is available remotely, study staff will obtain contact information from the present participants and will initiate a phone call. The study staff will then go through recruitment with the participant over the phone or via Webex.

*Incentives:* Families will receive the following incentives for their participation (participants approached in person will receive their incentive at the conclusion of a visit. Participants approached remotely will receive incentives via mail):

- Baseline: $5 meal card for each participant
- 1-month follow-up: $20 ClinCard for each participant
- 1-year follow-up: $30 ClinCard for each participant

*Retention:* Based on our previous work, engagement at 1-month post intervention is 100% due to patients actively being on treatment for their cancer diagnosis, routine clinical follow-up appointments, and other treatment-related in and outpatient encounters taking place at Nationwide Children’s Hospital. Therefore, we anticipate similarly high retention in this study. However, additional strategies will be used to keep families engaged throughout the study, including the 1-year post-intervention follow up. These include:

- Coordinating follow-up appointments with already scheduled clinic visits
- Routine correspondence with their medical team, and checks of the NCH electronic medical record for updated contact information or upcoming appointments within the hospital
- Check-in phone calls, emails, and/or text messages

# Withdrawal of Subjects*

Participants may be withdrawn if the intervention causes distress and it is determined by the interventionist and/or medical team that is not in the participant’s best interest to continue. If the participant withdrawals from the intervention, study staff will encourage completion of study data collection, per intent-to-treat methodology.

# Risks to Subjects*

It is expected that completing study questionnaires and interviews will introduce minimal risk to participants, yet the study team acknowledges that the new cancer diagnosis will be a stressor for AYAs and parents. Participants may experience some discomfort and/or embarrassment when completing survey items or interviews about FP. Given the study design, all patients and caregivers will have already received information about FP during the clinical fertility consult, per standard of care. Parents or AYAs may experience distress when asked about parent-child communication, although we do not expect this to have severe or long-lasting effects on the respondent. In preliminary work, parents and AYA have completed measures, similar to those in this study, within 10 minutes and provided positive feedback about the experience, emphasizing their perceived importance of addressing this topic. Loss of confidentiality is also a potential risk, although no more so than in any other research study. The study team will take every precaution to prevent this.

# Potential Benefits to Subjects*

Participants may or may not receive direct benefits from participating in this study. Questionnaires may help patients and families make FP decisions and the family-centered, psychoeducational decision tool may increase caregiver/child communication. This study also has strong potential to improve care for other AYA with cancer and their families, by optimizing FP rates and decision quality.

# Data Management* and Confidentiality

*Data Collection & Management:* Data collection will be the responsibility of the study staff under the supervision of the PI. The PI will be responsible for ensuring the accuracy, completeness, legibility, and timeliness of the data reported.

All source documents will be completed in a neat, legible manner to ensure accurate interpretation of data. Hardcopies of the study visit worksheets will be provided for use as source document worksheets for recording data for each participant consented/enrolled in the study. Data recorded in the electronic case report form (eCRF) derived from source documents will be consistent with the data recorded on the source documents.

Clinical data (including AEs) and clinical laboratory data will be entered into secure study databases, if needed. The data system includes password protection and internal quality checks, such as automatic range checks, to identify data that appear inconsistent, incomplete, or inaccurate. Clinical data will be entered directly from the source documents.

Only authorized study staff will have access to study data.

*Sample Size Determination:* The sample was determined based on the medium-large effects found in our previous work and work by Klosky et al. and is sufficient to find similar sized effects; thus our proposed sample size will enable us to detect effect sizes previously found in the literature^26,54-56^.

A sample size of *n*=20 per group will provide 80% power to detect large effect sizes of w=.45 for the chi-square analysis and d=.91 for the independent samples t-test. Because this is a pilot study, the focus will be on generating reliable effect size estimates for our primary outcome (FP uptake) comparing the intervention to standard care, rather than on statistical significance (using two-sided type I error rates (alpha values) of no more than .05). The proposed sample size is sufficient for gleaning this information.

*Data Analysis Plan:* Analyses will be conducted according to intent-to-treat methods, in which all randomized participants are included. Summary statistics will be calculated and qualitative methods used to evaluate the feasibility of recruiting and enrolling children.

Chi-squared and t-tests will be used to evaluate primary and secondary outcomes. A chi-square analyses will examine the primary outcome of FP uptake (Y/N) as a function of treatment group status An independent samples t-test will examine whether the secondary outcomes of decision quality (numerical score on the BSDQ and family communication (numerical score on the PACS and difference scores on the PACS for AYA and parents) change between 1-month and 1-year follow-ups. Pearson’s r correlations will be run to see if FP uptake (Y/N) is related to decision quality or family communication.

The two groups (intervention and control) will be examined in an *exploratory* manner to assess potential differences in factors known to affect FP decisions such as demographics (e.g. age), urgency to start treatment, knowledge, provider recommendation, and parent recommendation, as well as novel factors such as quality of parent-child communication. We will also examine these baseline factors as covariates within an analysis of covariance to see if might have a substantial impact on our effect size estimates for the larger RCT.

Quality Control of Data: Internal quality management of study conduct, data collection, documentation and completion will be conducted, under the direction of the PI. Quality control (QC) procedures will be implemented as follows:

- Informed consent: Study staff will review both the documentation of the consenting process as well as a percentage of the completed consent documents. This review will evaluate compliance with GCP, accuracy, and completeness. Feedback will be provided to the study team to ensure proper consenting procedures are followed.
- Source documents and the electronic data: Some data will be initially captured on source documents and will ultimately be entered into the study database. Some data will be entered directly into an electronic data capture system. To ensure accuracy of data initially captured on source documents, site staff will compare a representative sample of source data against the database, targeting key data points in that review. Some data will be entered directly into study databases. Electronic databases will be programed to alter study staff and/or participants of out of range values and/or potential errors.
- Protocol Deviations: The study team will review protocol deviations on an ongoing basis and will implement corrective actions when the quantity or nature of deviations are deemed to be at a level of concern. This protocol defines a protocol deviation as any noncompliance with the clinical trial protocol, International Council on Harmonization Good Clinical Practice (ICH GCP), or Manual of Procedures (MOP) requirements. The noncompliance may be either on the part of the participant, the investigator, or the study site staff. As a result of deviations, corrective actions will be developed by the site and implemented promptly, if needed. These practices are consistent with ICH GCP:
  - Section 4.5 Compliance with Protocol, subsections 4.5.1, 4.5.2, and 4.5.3
  - Section 5.1 Quality Assurance and Quality Control, subsection 5.1.1
  - Section 5.20 Noncompliance, subsections 5.20.1, and 5.20.2.

It is the responsibility of the PI to use continuous vigilance to identify and report deviations as required by the IRB and the funder, as well as oversee quality control of data. The site investigator is responsible for knowing and adhering to the reviewing IRB requirements. Should independent monitoring become necessary, the PI will provide direct access to all trial related sites, source data/documents, and reports for the purpose of monitoring and auditing by the funding agency, and inspection by local and regulatory authorities.

Handling of Data: The following data will be collected for each participant

- Demographics (e.g. age, race, ethnicity).
- Medical information (e.g. cancer type, treatment levels, infertility risk), collected via medical chart review.
- Information about decision and FP-related thoughts and feelings, collected via decision tool, parenthood goals surveys, andintervention.
- Participant report of communication with son or parents, collected via PACS survey.
- Participant report of decision satisfaction, collected via BSDQ.
- Participant report of feasibility and acceptability of participation in research.
- Open-ended interviews, recorded and transcribed verbatim. These interviews will focus on how participants made their decision, how they feel about their decision, alternatives they had considered, more information or support they would like to have had prior to their decision, what they would find helpful moving forward related to their fertility and future parenthood, and how participating in the study impacted them.

Study participant research data, which is for purposes of statistical analysis and scientific reporting, will be collected and maintained in REDCap, a secure electronic data capture system on the NCH research internet server. Some data may be collected on paper and will be entered into REDCap. Individual participants and their research data will be identified by a unique study identification number. Other types of data (e.g., audio recordings from the interviews and intervention) may be kept in a locked cabinet within a locked office at the hospital or on an NCH computer. Only direct study personnel will have access to this information. The study data entry, study management systems, and related study files will be secured and password protected.

Study documents will be retained for a minimum of 2 years after the formal discontinuation of clinical development of the study intervention. These documents will be retained for a longer period, if require by local regulations.

# Provisions to Monitor the Data to Ensure the Safety of Subjects*

Safety oversight for the study will be under the direction of the NCH IRB, as well as a Data Safety Monitoring Board (DSMB). The DSMB will consist of two faculty members from NCH. The first is a Professor of Pediatrics and Psychology at The Ohio State University and Director of the Biobehavioral Outcomes Core at NCH. She is a leading researcher in psychosocial outcomes among children and adolescents with significant health challenges, who has experience developing and executing intervention research with pediatric populations, as well as conducting multi-site studies involving complex multi-method longitudinal assessments. The second is an Associate Professor at The Ohio State University, an attending physician in Hematology/Oncology at NCH, director of the AYA Oncology Program, and a member of the Fertility and Reproductive Health team at NCH. He has been heavily involved in clinical research through the Children’s Oncology Group, specifically in the enrollment and management of patients on clinical trials.

The DSMB will be responsible for safeguarding the interests of trial participants, assessing the safety and efficacy of the intervention(s) during the trial, and for monitoring the overall conduct of the clinical trial. The DSMB will periodically review study results, evaluate the study interventions and procedures for adverse effects, and judge whether the overall integrity and conduct of the trial remain acceptable. Moreover, the DSMB may formulate and put forth recommendations to the study team, relating to the selection/recruitment/retention of participants and their management, and compliance to protocol specified regimens and the procedures for data management and quality control.

Summary reports regarding number of enrolled participants, and safety and risk (including any adverse events and unanticipated problems), will be generated at six-month intervals for the DSMB. Adverse events will be reported to the DSMB and IRB when they occur to determine if new procedures need to be implemented.

Safety information will be collected during routine interactions (phone calls, in-person visits, data collection timepoints) with participants.

Once the study begins, the study team will meet weekly to review the progress of the study (including information such as recruitment rate, reasons for declining participation, reasons for attrition), review data entry, assure data accuracy, detect potential errors at an early stage, and monitor staff compliance with training, regulatory, and confidentiality procedures.

The research will be suspended if the DSMB determines that is the best course of action, based on the data provided for review.

# Adverse Events & Serious Adverse Events

*Definitions:* This protocol defines an adverse event (AE) as any negative physical or emotional condition that was not present at baseline. This protocol defines a serious adverse event (SAE) as any AE that results in hospitalization, permanent disability, or death.

*Severity:* All AEs and SAEs are assessed by the principal investigator, and if necessary, another professional with clinical experience in the study population to determine their severity.

For AEs and SAEs, the following guidelines are used to describe severity:

- Mild: Events require minimal or no treatment and do not interfere with the participant’s daily activities.
- Moderate: Events result in a low level of inconvenience or concern with the therapeutic measures. Moderate events may cause some interference with functioning.
- Severe: Events interrupt a participant’s usual daily activity and may require systemic drug therapy or other treatment. Severe events are life-threatening or incapacitating.

*Relationship to the Intervention:* All AEs and SAEs are assessed by the principal investigator, and if necessary, another professional with clinical experience in the study population to determine their relationship to study intervention. The evaluation of relatedness considers etiologies such as natural history of the underlying disease, concurrent illness, concomitant therapy, study-related procedures, accidents, and other external factors.

The degree of certainty about causality is graded using the following categories:

- Related: The event is known to occur with the study procedures, there is a reasonable possibility that the study procedures caused the event, or there is a temporal relationship between the study procedures and the event. Reasonable possibility means that there is evidence to suggest a causal relationship between the study procedures and the event.
- Not Related: There is not a reasonable possibility that the study procedures caused the event, there is no temporal relationship between the study procedures and event onset, or an alternate etiology has been established.

*Expectedness:* All AEs and SAEs are assessed by the principal investigator, and if necessary, another professional with clinical experience in the study population to determine their expectedness. Expectedness is assessed based on the awareness of AEs and/or SAEs previously observed, not on the basis of what might be anticipated from the properties of the study intervention.

Expectedness of the event is assessed using the categories below:

- Expected: An event is considered expected if the nature, severity, or frequency of the event is consistent with the risk information previously described for the study procedures.
- Unexpected: An event is considered unexpected if the nature, severity, or frequency of the event is not consistent with the risk information previously described for the study procedures.

*Time Period and Frequency for Event Follow-up:* Research staff record events with occurring after informed consent is given up until the end of the intervention. The occurrence of AEs and/or SAEs may come to the attention of study personnel during study visits, interviews, and questionnaires; or when a study participant presents for medical care.

AEs and SAEs are captured on the appropriate case report form (CRF). Information collected includes event description; time of onset; clinician assessment of severity, relationship to study procedures (assessed only by those with the training and authority to make a diagnosis), expectedness; and time of resolution/stabilization of the event. All AEs and SAEs occurring during the above-mentioned timeframe are documented appropriately regardless of relationship to the intervention. Events are followed for outcome information until resolution or stabilization.

*Reporting:* AEs are reported to the NCH IRB at least annually. AEs are reported to the DSMB at each meeting. AEs are reported to NCI according to their recommended timelines. Per NCH HRP-103 (updated 12/10/18), SAEs determined to be related to the intervention and unexpected are reported to the NCH IRB within 5 business days of discovery; other SAEs are reported to the NCH IRB annually. SAEs are reported to the DSMB and to NCI according to their recommended timelines.

# Unanticipated Problems

*Definition:* This protocol uses the definition of Unanticipated Problems as defined by the Office for Human Research Protections (OHRP). OHRP considers unanticipated problems involving risks to participants or others to include, in general, any incident, experience, or outcome that meets **all** the following criteria:

- Unexpected in terms of nature, severity, or frequency given (a) the research procedures that are described in the protocol-related documents, such as the Institutional Review Board (IRB)-approved research protocol and informed consent document; and (b) the characteristics of the participant population being studied;
- Related or possibly related to participation in the research ("possibly related" means there is a reasonable possibility that the incident, experience, or outcome may have been caused by the procedures involved in the research); and
- Suggests that the research places participants or others at a greater risk of harm (including physical, psychological, economic, or social harm) than was previously known or recognized.

*Reporting:* Unanticipated problems (UPs) are reported by the PI to the Institutional Review Board (IRB) and the DSMB within 5 business days of discovery. SAEs are initially reported to NCI within five calendar days, with a full report filed within 10 calendar days.

# Provisions to Protect the Privacy Interests of Subjects

*Plan to Protect Participant Privacy:* Participant confidentiality and privacy is strictly held in trust by the participating investigators, their staff, the safety and oversight monitor(s), and the funding agency. This confidentiality is extended to the data being collected as part of this study. Data that could be used to identify a specific study participant will be held in strict confidence within the research team. No personally-identifiable information from the study will be released to any unauthorized third party without prior written approval of the funding agency.

All research activities will be conducted in as private a setting, as much as possible.

Authorized representatives of the sponsor, study monitors, representatives of the Institutional Review Board (IRB), or regulatory agencies may inspect all documents and records required to be maintained by the investigator. The clinical study site will permit access to such records.

The study participant’s contact information will be securely stored for internal use during the study. At the end of the study, all records will continue to be kept in a secure location for as long a period as dictated by the reviewing IRB, Institutional policies, or other requirements.

Study participant research data, which is for purposes of statistical analysis and scientific reporting, will be collected and maintained in REDCap, a secure electronic data capture system on the NCH research internet server. Individual participants and their research data will be identified by a unique study identification number. Other types of data (e.g., audio recordings from the interviews and intervention, paper surveys) may be kept in a locked cabinet within a locked office at the hospital or on an NCH computer. Interviews will be audio recorded and transcribed verbatim. Both the audio files and transcriptions will be stored on a secured intranet server only accessible by research staff. All identifying information will be removed from the transcripts and audio files will be deleted after data collection is completed. Only direct study personnel will have access to this information. The study data entry, study management systems, and related study files will be secured and password protected.

To help participants feel at ease and able to speak openly, study staff will be trained in active interviewing techniques (e.g. active listening, empathizing, proper body language). After interviews, staff will debrief with the principal investigators and address any issues that may have arisen during the interview.

To further protect the privacy of study participants, the Secretary, Health and Human Services (HHS), has issued a Certificate of Confidentiality (CoC) to all researchers engaged in biomedical, behavioral, clinical or other human subjects research funded wholly or in part by the federal government. Recipients of NIH funding for human subjects research are required to protect identifiable research information from forced disclosure per the terms of the NIH Policy (see https://humansubjects.nih.gov/coc/index). As set forth in 45 CFR Part 75.303(a) and NIHGPS Chapter 8.3, recipients conducting NIH-supported research covered by this Policy are required to establish and maintain effective internal controls (e.g., policies and procedures) that provide reasonable assurance that the award is managed in compliance with Federal statutes, regulations, and the terms and conditions of award. It is the NIH policy that investigators and others who have access to research records will not disclose identifying information except when the participant consents or in certain instances when federal, state, or local law or regulation requires disclosure. NIH expects investigators to inform research participants of the protections and the limits to protections provided by a Certificate issued by this Policy.

# Compensation for Research-Related Injury

N/A

# Economic Burden to Subjects

N/A

# Consent Process

Consent forms describing in detail the study intervention, study procedures, and risks are given to the participant and written documentation of informed consent is required prior to starting any data collection procedures or randomizing the family. Families will be given a copy of the signed consent form for their records.

Caregivers will provide informed consent for their children aged 12-17 years, as well as themselves. Child participants who are 18 years old or older will provide consent for themselves. Children, aged 12-17, will additionally provide assent. Informed consent and assent (if applicable) will be obtained, prior to any study procedures taking place. During the process, the study will be described to participants and families will have the opportunity to discuss participation, before enrolling. Throughout the study, participants will be reminded of the voluntary nature of their involvement.

Consent will take place either in-person or via remote connection. In-person participants will each receive a copy of the consent form to follow along as the study staff reviews its content. The study staff will then collect all participants’ signatures on a single form. Consent will be obtained from remote participant(s) by sending an electronic REDCap link via email. During the process, participants will be connected in via phone or Webex and will have the opportunity to discuss participation. Participants will be provided with an electronic copy of the consent form after submitting the REDCap version.

Trained study staff will obtain consent according to IRB policy, SOP: Informed Consent Process for Research (HRP-090) to ensure participants understand and do not feel coerced into consenting.

**Waiver of Written Documentation of Consent (verbal)**

N/A

# Process to Document Consent in Writing

Consent forms describing in detail the study intervention, study procedures, and risks are given to the participant and written documentation of informed consent is required prior to starting any data collection procedures or randomizing the family. Families will be given a copy of the signed consent form for their records.

# Setting

Research will be conducted at a single-site, Nationwide Children’s Hospital (Columbus, OH). After an initial and new cancer diagnosis, an automatic referral is placed for a fertility consult and Dr. Nahata is immediately notified of the age and sex of the patient. The patient and family then consult with a fertility navigator to discuss fertility options, per standard of care. During the fertility consult the fertility navigator will assess pubertal status/eligibility for sperm banking, provide verbal and written information about infertility risk and FP options (masturbation/sperm extraction), and then introduce the study. The patient’s eligibility is communicated to study staff who then approach the family for recruitment purposes.

All study procedures except interviews will take place in the patient’s hospital room (or clinic room), or other private location at Nationwide Children’s Hospital. Interviews will be carried in private consultation rooms on the treatment floors, or other private location at Nationwide Children’s Hospital. In the event that a parent (or the entire family) is not present, surveys or interviews may be administered over the phone or via Webex.

# Resources Available

Feasibility: There are approximately 30 males newly diagnosed with cancer in our target age range each year. Taking into account our inclusion/exclusion criteria, infrastructure of the fertility consult service, and recruitment rates in our pilot (>90%), we anticipate completing our recruitment goals within the allocated timeframe.

*Time Devoted to the Research:* Dr. Nahata assembled a multidisciplinary team with expertise in mixed methods research; family systems and communication; and the design, implementation, and analysis of clinical trials to support and carry out this project.

As the Principal Investigator of the project, Dr. Nahata will assume overall responsibility for the administrative and scientific conduct of the study. Dr. Nahata will maintain the consistency and integrity of study materials and training related to data collection. She will submit and gain IRB approval for the RCT and will coordinate, conduct and/or supervise all aspects of the recruitment process, data collection, data management, statistical and qualitative analysis, and dissemination of results. As the medical director of the NCH Fertility and Reproductive Health team, Dr. Nahata is immediately notified about every new fertility consult that is placed, which has led to >90% recruitment rates in the pilot.

Dr. Gerhardt will provide focused guidance on 1) designing the facilitated conversation prompts, 2) conducting the intervention, 3) coding and thematic analysis, 4) using qualitative data to enhance quantitative data, and 5) integrating quantitative and qualitative data from multiple informants into a statistical model.

Dr. O’Brien will provide guidance on how to sensitively approach AYA newly diagnosed with cancer and their families to optimize recruitment and retention, as well as direct mentorship in RCT design and implementation.

Dr. Quinn will provide mentorship in adapting this tool to our study population. She will assist with the qualitative analyses and provide input in developing the family-centered psychoeducational intervention. Dr. Rausch will oversee data management and analyses.

Dr. Klosky will provide guidance on the content of the intervention.

Research assistants will assist with recruitment and data collection, and an individual with ENRICH/ECHO (oncofertility communication) training will deliver the intervention.

*Facilities:* All study procedures except interviews will take place in the patient’s hospital room (or clinic room), or other private location at Nationwide Children’s Hospital. Private consultation rooms are available on the treatment floors, or other private location at Nationwide Children’s Hospital.

*Availability of Medical or Psychological Resources:* As part of their cancer treatment, patients are assigned a psychosocial provider who can be called as needed. Additionally, participants are being treated by an interdisciplinary medical team.

Process to ensure that all persons assisting with the research are adequately informed about the protocol, the research procedures, and their duties and functions: All study personnel who will be working with data or protected health information will be properly trained, which includes completing the online Collaborative Institutional Training Initiative (CITI) certification and undergoing a day-long instructional seminar at the hospital for protecting human subjects and engaging in best practices for responsible conduct of research. Additionally, per NIH policy, clinical trial staff who are involved in the design, conduct, oversight, or management of the trial will be trained in Good Clinical Practice (GCP). Additionally, staff will receive extensive training in administration of all questionnaires as well as professional behavior with families. The interventionist will have additional training in ENRICH/ECHO, an oncofertility communication training program. Finally, Staff will be trained to contact the PI immediately if an adverse event occurs, and corrective action, including IRB notification, will occur, if necessary.

# Multi-Site Research*

N/A

# Protected Health Information Recording

- 1. **Indicate which subject identifiers will be recorded for this research.**

Name

Complete Address

Telephone or Fax Number

Social Security Number (do not check if only used for ClinCard)

Dates (treatment dates, birth date, date of death)

Email address, IP address or URL

Medical Record Number or other account number

Health Plan Beneficiary Identification Number

Full face photographic images and/or any comparable images (x-rays)

Account Numbers

Certificate/License Numbers

Vehicle Identifiers and Serial Numbers (e.g. VINs, License Plate Numbers)

Device Identifiers and Serial Numbers

Biometric identifiers, including finger and voice prints

Other number, characteristic or code that could be used to identify an individual

None (Complete De-identification Certification Form)

- 1. **Check the appropriate category and attach the required form* on the Local Site Documents, #3. Other Documents, page of the application.  (Choose one.)**

Patient Authorization will be obtained. (Include the appropriate HIPAA language (see Section 14 of consent template) in the consent form OR attach the HRP-900, HIPAA AUTHORIZATION form.)

Protocol meets the criteria for waiver of authorization. (Attach the HRP-901, WAIVER OF HIPAA AUTHORIZATION REQUEST form.)

Protocol is using de-identified information. (Attach the HRP-902, DE-IDENTIFICATION CERTIFICATION form.) (Checked "None" in 1.0 above)

Protocol involves research on decedents. (Attach the HRP-903, RESEARCH ON DECEDENTS REQUEST form.)

Protocol is using a limited data set and data use agreement. (Contact the Office of Technology Commercialization to initiate a Limited Data Use Agreement.

# ****How long will identifying information on each participant be maintained?****

The PHI collected or created under this research study will be used or shared as needed until the end of the study. The records of this study will be kept for an indefinite period of time and your authorization to use or share your PHI will not expire.

# ****Describe any plans to code identifiable information collected about each participant.****

**Once enrolled in the study, participants (patients and parent(s)) will receive a study ID number. This number will be entered on their survey, be it paper or online (i.e., using REDCap, an online survey system that is housed securely on the NCH research drive). The only link between the participant’s name and study ID will be an electronic tracking sheet, which will be password protected and stored on the secured research network drive of the hospital. The tracking sheet will contain the following protected health information: name, medical record number, date of birth, address, phone number, diagnosis, and dates of study visits. Only direct study personnel will have access to this file. Participants will be identified in all datasets by their study ID.**

- 1. **Check each box that describes steps that will be taken to safeguard the confidentiality of information collected for this research:**

Research records will be stored in a locked cabinet in a secure location

Research records will be stored in a password-protected computer file

The list linking the assigned code number to the individual subject will be maintained separately from the other research data

Only certified research personnel will be given access to identifiable subject information

- 1. **Describe the provisions included in the protocol to protect the privacy interests of subjects, where "privacy interests" refer to the interest of individuals in being left alone, limiting access to them, and limiting access to their information. (This is not the same provision to maintain the confidentiality of data.)**

**Participants may opt out of participating in this study at any time**. If a participant or family requests to be removed from the study, study staff will cease further contact with the family. If a patient declines to participate in the study, no further information will be gathered.

# Confidential Health Information

- 1. **Please mark all categories that reflect the nature of health information to be accessed and used as part of this research.**

Demographics (age, gender, educational level)

Diagnosis

Laboratory reports

Radiology reports

Discharge summaries

Procedures/Treatments received

Dates related to course of treatment (admission, surgery, discharge)

Billing information

Names of drugs and/or devices used as part of treatment

Location of treatment

Name of treatment provider

Surgical reports

Other information related to course of treatment

None

# Please discuss why it is necessary to access and review the health information noted in your response above.

Medical information for each patient will be accessed to ensure eligibility and to provide data for analysis on factors that impact FP decisions and decision satisfaction.

# Is the health information to be accessed and reviewed the minimal necessary to achieve the goals of this research? Yes No

# Will it be necessary to record information of a sensitive nature? Yes No

# Do you plan to obtain a federally-issued Certificate of Confidentiality as a means of protecting the confidentiality of the information collected? Yes No

# References

1. Ward E, DeSantis C, Robbins A, Kohler B, Jemal A. Childhood and adolescent cancer statistics, 2014. *CA: a cancer journal for clinicians.* 2014;64(2):83-103.

2. Phillips SM, Padgett LS, Leisenring WM, et al. Survivors of childhood cancer in the United States: prevalence and burden of morbidity. *Cancer epidemiology, biomarkers & prevention : a publication of the American Association for Cancer Research, cosponsored by the American Society of Preventive Oncology.* 2015;24(4):653-663.

3. Green DM, Liu W, Kutteh WH, et al. Cumulative alkylating agent exposure and semen parameters in adult survivors of childhood cancer: a report from the St Jude Lifetime Cohort Study. *Lancet Oncol.* 2014;15(11):1215-1223.

4. Brignardello E, Felicetti F, Castiglione A, et al. Endocrine health conditions in adult survivors of childhood cancer: the need for specialized adult-focused follow-up clinics. *European journal of endocrinology / European Federation of Endocrine Societies.* 2013;168(3):465-472.

5. Green DM, Kawashima T, Stovall M, et al. Fertility of male survivors of childhood cancer: a report from the Childhood Cancer Survivor Study. *Journal of clinical oncology : official journal of the American Society of Clinical Oncology.* 2010;28(2):332-339.

6. Stinson JN, Jibb LA, Greenberg M, et al. A Qualitative Study of the Impact of Cancer on Romantic Relationships, Sexual Relationships, and Fertility: Perspectives of Canadian Adolescents and Parents During and After Treatment. *Journal of adolescent and young adult oncology.* 2015;4(2):84-90.

7. Loren AW, Mangu PB, Beck LN, et al. Fertility preservation for patients with cancer: American Society of Clinical Oncology clinical practice guideline update. *Journal of clinical oncology : official journal of the American Society of Clinical Oncology.* 2013;31(19):2500-2510.

8. Chong AL, Gupta A, Punnett A, Nathan PC. A cross Canada survey of sperm banking practices in pediatric oncology centers. *Pediatric blood & cancer.* 2010;55(7):1356-1361.

9. Schover LR, Brey K, Lichtin A, Lipshultz LI, Jeha S. Knowledge and experience regarding cancer, infertility, and sperm banking in younger male survivors. *Journal of clinical oncology : official journal of the American Society of Clinical Oncology.* 2002;20(7):1880-1889.

10. Bann CM, Treiman K, Squiers L, et al. Cancer Survivors' Use of Fertility Preservation. *J Womens Health (Larchmt).* 2015;24(12):1030-1037.

11. Benedict C, Shuk E, Ford JS. Fertility Issues in Adolescent and Young Adult Cancer Survivors. *Journal of adolescent and young adult oncology.* 2016;5(1):48-57.

12. Grover NS, Deal AM, Wood WA, Mersereau JE. Young Men With Cancer Experience Low Referral Rates for Fertility Counseling and Sperm Banking. *Journal of oncology practice / American Society of Clinical Oncology.* 2016;12(5):465-471.

13. Nilsson J, Jervaeus A, Lampic C, et al. 'Will I be able to have a baby?' Results from online focus group discussions with childhood cancer survivors in Sweden. *Hum Reprod.* 2014;29(12):2704-2711.

14. Ellis SJ, Wakefield CE, McLoone JK, Robertson EG, Cohn RJ. Fertility concerns among child and adolescent cancer survivors and their parents: A qualitative analysis. *Journal of psychosocial oncology.* 2016;34(5):347-362.

15. Stein DM, Victorson DE, Choy JT, et al. Fertility Preservation Preferences and Perspectives Among Adult Male Survivors of Pediatric Cancer and Their Parents. *Journal of adolescent and young adult oncology.* 2014;3(2):75-82.

16. Armuand GM, Wettergren L, Rodriguez-Wallberg KA, Lampic C. Desire for children, difficulties achieving a pregnancy, and infertility distress 3 to 7 years after cancer diagnosis. *Supportive care in cancer : official journal of the Multinational Association of Supportive Care in Cancer.* 2014;22(10):2805-2812.

17. Gorman JR, Su HI, Pierce JP, Roberts SC, Dominick SA, Malcarne VL. A multidimensional scale to measure the reproductive concerns of young adult female cancer survivors. *Journal of cancer survivorship : research and practice.* 2014;8(2):218-228.

18. Peate M, Meiser B, Cheah BC, et al. Making hard choices easier: a prospective, multicentre study to assess the efficacy of a fertility-related decision aid in young women with early-stage breast cancer. *British journal of cancer.* 2012;106(6):1053-1061.

19. Chow EJ, Stratton KL, Leisenring WM, et al. Pregnancy after chemotherapy in male and female survivors of childhood cancer treated between 1970 and 1999: a report from the Childhood Cancer Survivor Study cohort. *Lancet Oncol.* 2016;17(5):567-576.

20. Crawshaw MA, Glaser AW, Hale JP, Sloper P. Young males' experiences of sperm banking following a cancer diagnosis - a qualitative study. *Human fertility.* 2008;11(4):238-245.

21. Sheth KR, Sharma V, Helfand BT, et al. Improved fertility preservation care for male patients with cancer after establishment of formalized oncofertility program. *The Journal of urology.* 2012;187(3):979-986.

22. Kelvin JF, Thom B, Benedict C, et al. Cancer and Fertility Program Improves Patient Satisfaction With Information Received. *Journal of clinical oncology : official journal of the American Society of Clinical Oncology.* 2016;34(15):1780-1786.

23. Shnorhavorian M, Kroon L, Jeffries H, Johnson R. Creating a standardized process to offer the standard of care: continuous process improvement methodology is associated with increased rates of sperm cryopreservation among adolescent and young adult males with cancer. *Journal of pediatric hematology/oncology.* 2012;34(8):e315-319.

24. Klosky JL, Anderson LE, Russell KM, et al. Provider Influences on Sperm Banking Outcomes Among Adolescent Males Newly Diagnosed With Cancer. *The Journal of adolescent health : official publication of the Society for Adolescent Medicine.* 2017;60(3):277-283.

25. Klosky JL SJ, Lehmann V, Russell KM, Wang F, Hardin RN, Eddinger JR, Zhang H, Dahlke LAM, Schover LR. Parental influences on sperm banking attempts among adolescent males newly diagnosed with cancer. *Fertility and sterility.*

26. Klosky JL, Wang F, Russell KM, et al. Prevalence and Predictors of Sperm Banking in Adolescents Newly Diagnosed With Cancer: Examination of Adolescent, Parent, and Provider Factors Influencing Fertility Preservation Outcomes. *Journal of clinical oncology : official journal of the American Society of Clinical Oncology.* 2017:JCO2016704767.

27. Klosky JL, Simmons JL, Russell KM, et al. Fertility as a priority among at-risk adolescent males newly diagnosed with cancer and their parents. *Supportive care in cancer : official journal of the Multinational Association of Supportive Care in Cancer.* 2015;23(2):333-341.

28. Quinn GP, Knapp C, Murphy D, Sawczyn K, Sender L. Congruence of reproductive concerns among adolescents with cancer and parents: pilot testing an adapted instrument. *Pediatrics.* 2012;129(4):e930-936.

29. Nahata L, Morgan TL, Lipak KG, et al. Perceptions of participating in family-centered fertility research among adolescent and young adult males newly diagnosed with cancer: A qualitative study. *Pediatric blood & cancer.* 2019;66(11):e27966.

30. Hudson MM, Ness KK, Gurney JG, et al. Clinical ascertainment of health outcomes among adults treated for childhood cancer. *Jama.* 2013;309(22):2371-2381.

31. Kenney LB, Cohen LE, Shnorhavorian M, et al. Male reproductive health after childhood, adolescent, and young adult cancers: a report from the Children's Oncology Group. *Journal of clinical oncology : official journal of the American Society of Clinical Oncology.* 2012;30(27):3408-3416.

32. Oktay K, Harvey BE, Partridge AH, et al. Fertility Preservation in Patients With Cancer: ASCO Clinical Practice Guideline Update. *Journal of clinical oncology : official journal of the American Society of Clinical Oncology.* 2018:JCO2018781914.

33. Kenney LB, Laufer MR, Grant FD, Grier H, Diller L. High risk of infertility and long term gonadal damage in males treated with high dose cyclophosphamide for sarcoma during childhood. *Cancer.* 2001;91(3):613-621.

34. Nahata L, Cohen LE, Lehmann LE, Yu RN. Semen analysis in adolescent cancer patients prior to bone marrow transplantation: when is it too late for fertility preservation? *Pediatric blood & cancer.* 2013;60(1):129-132.

35. Kenney LB, Duffey-Lind E, Ebb D, Sklar CA, Grier H, Diller L. Impaired testicular function after an ifosfamide-containing regimen for pediatric osteosarcoma: a case series and review of the literature. *Journal of pediatric hematology/oncology.* 2014;36(3):237-240.

36. Lehmann V, Keim MC, Nahata L, et al. Fertility-related knowledge and reproductive goals in childhood cancer survivors: short communication. *Hum Reprod.* 2017;32(11):2250-2253.

37. Nahata L, Cohen LE, Yu RN. Barriers to fertility preservation in male adolescents with cancer: it's time for a multidisciplinary approach that includes urologists. *Urology.* 2012;79(6):1206-1209.

38. Flink DM, Sheeder J, Kondapalli LA. A Review of the Oncology Patient's Challenges for Utilizing Fertility Preservation Services. *Journal of adolescent and young adult oncology.* 2016.

39. Panagiotopoulou N, van Delft FW, Hale JP, Stewart JA. Fertility Preservation Care for Children and Adolescents with Cancer: An Inquiry to Quantify Professionals' Barriers. *Journal of adolescent and young adult oncology.* 2017;6(3):422-428.

40. Campbell JE, Assanasen C, Robinson RD, Knudtson JF. Fertility Preservation Counseling for Pediatric and Adolescent Cancer Patients. *Journal of adolescent and young adult oncology.* 2016;5(1):58-63.

41. Tam S, Puri N, Stephens D, et al. Improving Access to Standardized Fertility Preservation Information for Older Adolescents and Young Adults with Cancer: Using a User-Centered Approach with Young Adult Patients, Survivors, and Partners to Refine Fertility Knowledge Transfer. *Journal of cancer education : the official journal of the American Association for Cancer Education.* 2016.

42. Champion VL. Revised susceptibility, benefits, and barriers scale for mammography screening. *Research in nursing & health.* 1999;22(4):341-348.

43. Janz NK, Becker MH. The Health Belief Model: a decade later. *Health Educ Q.* 1984;11(1):1-47.

44. Champion VL. Strategies to increase mammography utilization. *Medical care.* 1994;32(2):118-129.

45. Ter Keurst A, Boivin J, Gameiro S. Women's intentions to use fertility preservation to prevent age-related fertility decline. *Reproductive biomedicine online.* 2016;32(1):121-131.

46. Fulford B, Bunting L, Tsibulsky I, Boivin J. The role of knowledge and perceived susceptibility in intentions to optimize fertility: findings from the International Fertility Decision-Making Study (IFDMS). *Hum Reprod.* 2013;28(12):3253-3262.

47. Ginsberg JP, Ogle SK, Tuchman LK, et al. Sperm banking for adolescent and young adult cancer patients: sperm quality, patient, and parent perspectives. *Pediatric blood & cancer.* 2008;50(3):594-598.

48. Li N, Jayasinghe Y, Kemertzis MA, Moore P, Peate M. Fertility Preservation in Pediatric and Adolescent Oncology Patients: The Decision-Making Process of Parents. *Journal of adolescent and young adult oncology.* 2017;6(2):213-222.

49. Klosky JL, Flynn JS, Lehmann V, et al. Parental influences on sperm banking attempts among adolescent males newly diagnosed with cancer. *Fertility and sterility.* 2017;108(6):1043-1049.

50. Crawshaw MA, Glaser AW, Pacey AA. The use of pornographic materials by adolescent male cancer patients when banking sperm in the UK: legal and ethical dilemmas. *Human fertility.* 2007;10(3):159-163.

51. de Vries MC, Bresters D, Engberts DP, Wit JM, van Leeuwen E. Attitudes of physicians and parents towards discussing infertility risks and semen cryopreservation with male adolescents diagnosed with cancer. *Pediatric blood & cancer.* 2009;53(3):386-391.

52. Galvin KM, Clayman ML. Whose Future Is It? Ethical Family Decision Making About Daughters' Treatment in the Oncofertility Context. *Cancer Treat Res.* 2010;156:429-445.

53. Nahata L MT, Caltabellotta N, Yeager N, Ferrante A, Rausch J, Quinn G, O'Brien S, Gerhardt C. Congruence of reproductive goals and concerns among male adolescent and young adult cancer survivors and their parents. *In Preparation.* 2018.

54. Chen HN, Cohen P, Chen S. How Big is a Big Odds Ratio? Interpreting the Magnitudes of Odds Ratios in Epidemiological Studies. *Commun Stat-Simul C.* 2010;39(4):860-864.

55. Hsieh FY. Sample size tables for logistic regression. *Statistics in medicine.* 1989;8(7):795-802.

56. Ferrante AC GC, Yeager ND, Rausch JR, Lehmann V, O'Brien SH, Quinn GP, Nahata L. Factors Related to Interest in Learning about Fertility Status among AYA Male Cancer Survivors. *In Preparation.* 2018.
